# Transcriptomic analysis of aggressive meningiomas identifies PTTG1 and LEPR as prognostic biomarkers independent of WHO grade

**DOI:** 10.18632/oncotarget.7396

**Published:** 2016-02-15

**Authors:** Melissa Schmidt, Andreas Mock, Christine Jungk, Felix Sahm, Anna Theresa Ull, Rolf Warta, Katrin Lamszus, Konstantinos Gousias, Ralf Ketter, Saskia Roesch, Carmen Rapp, Sebastian Schefzyk, Steffi Urbschat, Bernd Lahrmann, Almuth F. Kessler, Mario Löhr, Christian Senft, Niels Grabe, David Reuss, Philipp Beckhove, Manfred Westphal, Andreas von Deimling, Andreas Unterberg, Matthias Simon, Christel Herold-Mende

**Affiliations:** ^1^ Division of Experimental Neurosurgery, Department of Neurosurgery, University of Heidelberg, Heidelberg, Germany; ^2^ Department of Neuropathology, Heidelberg University Hospital, CCU Neuropathology, German Consortium for Translational Cancer Research (DKTK), German Cancer Research Center (DKFZ), Heidelberg, Germany; ^3^ Department of Neurosurgery, University Medical Center Hamburg-Eppendorf, Hamburg, Germany; ^4^ Department of Neurosurgery, University Hospital Bonn, Bonn, Germany; ^5^ Department of Neurosurgery, Saarland University, Medical School, Homburg, Germany; ^6^ Bioquant, Medical Oncology, National Center for Tumor Diseases, Heidelberg, Germany; ^7^ Department of Neurosurgery, University Hospital of Würzburg, Würzburg, Germany; ^8^ Department of Neurosurgery, University of Frankfurt, Frankfurt, Germany; ^9^ Regensburg Center for Interventional Immunology, RCI and University Medical Center of Regensburg, Regensburg, Germany

**Keywords:** meningioma, anaplastic, recurrent, transcriptomic analysis, biomarker

## Abstract

Meningiomas are frequent central nervous system tumors. Although most meningiomas are benign (WHO grade I) and curable by surgery, WHO grade II and III tumors remain therapeutically challenging due to frequent recurrence. Interestingly, relapse also occurs in some WHO grade I meningiomas. Hence, we investigated the transcriptional features defining aggressive (recurrent, malignantly progressing or WHO grade III) meningiomas in 144 cases. Meningiomas were categorized into non-recurrent (NR), recurrent (R), and tumors undergoing malignant progression (M) in addition to their WHO grade. Unsupervised transcriptomic analysis in 62 meningiomas revealed transcriptional profiles lining up according to WHO grade and clinical subgroup. Notably aggressive subgroups (R+M tumors and WHO grade III) shared a large set of differentially expressed genes (n=332; p<0.01, FC>1.25). In an independent multicenter validation set (n=82), differential expression of 10 genes between WHO grades was confirmed. Additionally, among WHO grade I tumors differential expression between NR and aggressive R+M tumors was affirmed for *PTTG1, AURKB, ECT2, UBE2C* and *PRC1,* while *MN1* and *LEPR* discriminated between NR and R+M WHO grade II tumors. Univariate survival analysis revealed a significant association with progression-free survival for *PTTG1*, *LEPR, MN1, ECT2, PRC1, COX10, UBE2C* expression, while multivariate analysis identified a prediction for *PTTG1* and *LEPR* mRNA expression independent of gender, WHO grade and extent of resection. Finally, stainings of PTTG1 and LEPR confirmed malignancy-associated protein expression changes. In conclusion, based on the so far largest study sample of WHO grade III and recurrent meningiomas we report a comprehensive transcriptional landscape and two prognostic markers.

## INTRODUCTION

Meningiomas are common brain tumors accounting for approximately 36 % of all primary central nervous system tumors [1]. Based on histopathological criteria, meningiomas are classified into three WHO grades [2]. Around 80% belong to the benign WHO°I, 15-20 % are classified as atypical WHO°II and 1-2 % as anaplastic WHO°III meningiomas [3,4]. Even after aggressive resection tumor recurrence may occur, but recurrence rates vary substantially between WHO grades. While only 5 % of all completely resected (Simpson grade 1-3) WHO°I meningiomas relapse within 5 years, the 5-year recurrence rate for atypical meningioma WHO°II is about 40 %, and for malignant meningiomas WHO°III as high as 50-80 % [4–6]. Although anaplastic meningiomas show a low prevalence, they constitute the most aggressive and the most therapeutically challenging subgroup. Despite current standard therapy consisting of maximum tumor resection followed by radiotherapy [7], the prognosis for anaplastic meningiomas WHO°III remains dismal with a median overall survival of only 1.5 years [5]. Tumor relapse and its treatment is a major source of morbidity (and even mortality), and in some patients recurrent meningiomas even undergo a malignant progression to a higher WHO grade than the primary tumor [5]. To date, higher WHO grade, incomplete tumor resection (Simpson grade ≥4) as well as a high Ki67 index have been associated with recurrence [8–10]. However, the accuracy of predicting recurrence even in higher-grade meningioma based on these criteria is still insufficient. Therefore, novel prognostic factors are needed to predict the risk for relapse, and thus to estimate which patients might need a more intense therapy and shorter follow-up intervals.

While comprehensive transcriptional landscapes are already guiding more accurate prediction of patient survival and the development of targeted therapies in a multitude of tumor diseases [11,12], molecular analyses in meningioma are still far from clinical application. Nevertheless, meningiomas have been characterized by a set of chromosomal abnormalities [13]. Partial loss of chromosome 22 has been associated with tumorigenesis and represents the most common genetic alteration in meningiomas of all WHO grades [14,15]. Moreover, deletions in chromosomes 1p, 10q and 14q, and chromosomal gains on 1q, 9p, 12q, 15q, 17q, and 20q are frequent alterations in WHO°II and °III meningiomas [3,14,16]. Furthermore, anaplastic (WHO°III) meningiomas display genetic losses of 6q, 9p 10q, 14q and amplifications on 17q23 [17,18]. More recently, advanced genomic analyses identified mutations in the *TRAF7, KLF4, AKT1* and *SMO* genes as common aberrations in meningiomas [19–22]. In addition to these genetic studies, several transcriptomic analyses were performed with the aim to identify progression-associated genes [23–28] [29]. However, congruency is minimal and comparisons suffer from small study sample sizes especially in WHO°III [28] and recurrent meningiomas [24].

In contrast, in the present multicenter study we performed gene expression analyses in altogether 144 meningiomas including an extraordinary high number of 59 WHO°III tumors and a substantial number of WHO°I and °II tumors with a dismal clinical course. This allowed for the identification of 10 genes with differential expression between WHO grades. Among these, *PTTG1* and *LEPR* showed a significant association with recurrence independent of known prognostic confounders such as WHO grade and therefore might serve in the future as novel putative biomarkers to predict aggressiveness of meningiomas.

## PATIENTS AND METHODS

### Study samples

**Figure 1:**
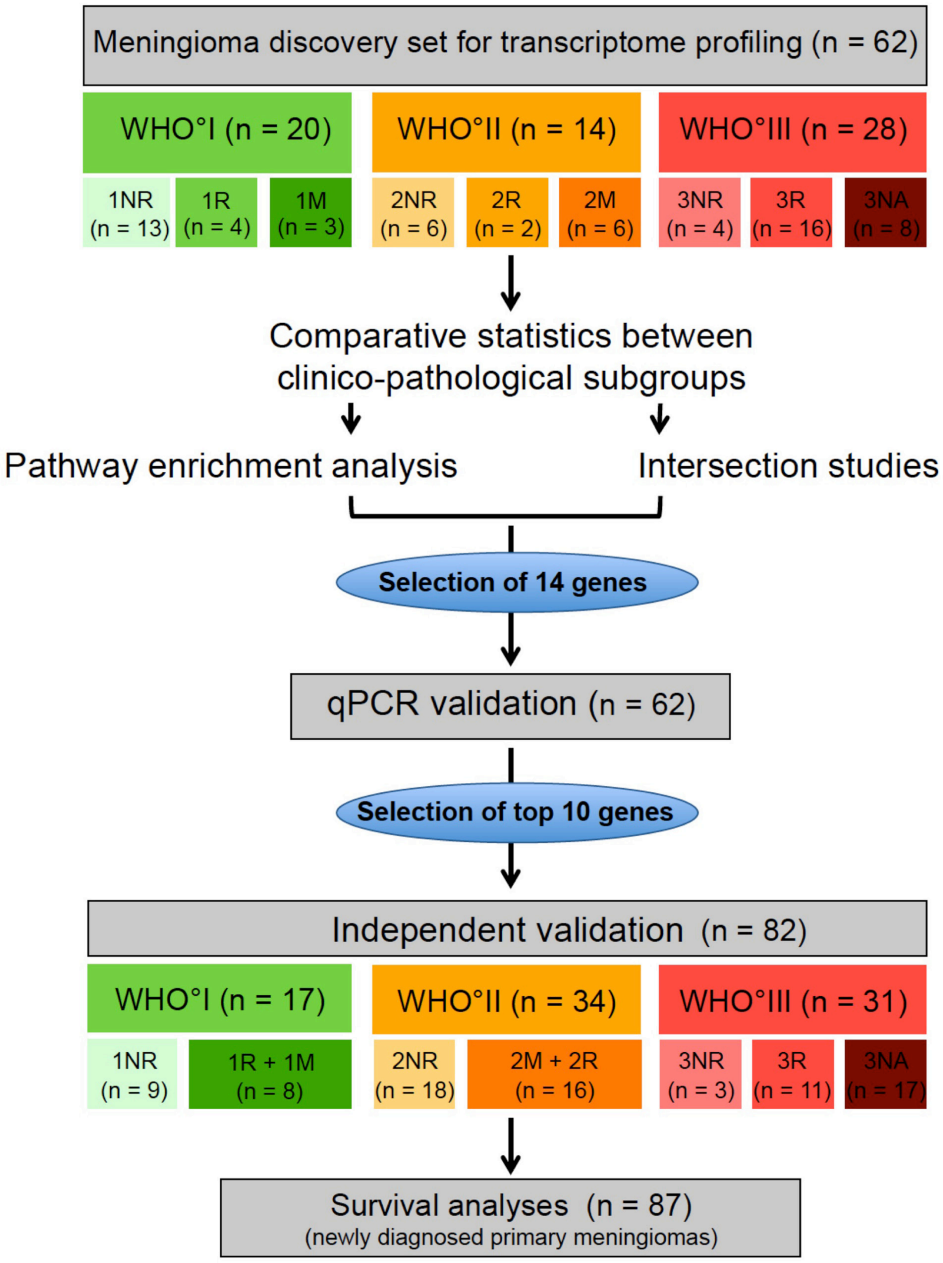
Study design Transcriptome profiling was performed in a microarray discovery set (n = 62). Meningiomas of the microarray set were categorized according to their WHO grade and their future clinical behavior: NR = non recurrent (meningioma without future recurrence), R = recurrent (meningioma with future recurrence of the same WHO grade), M = tumors with malignant progression (meningioma with future recurrence as higher WHO grade), NA = no available clinical data for classification. Based on comparative statistics between the clinico-pathological subgroups, pathway enrichment analysis and intersection analysis, 14 genes were selected for technical qPCR validation. 10 genes, that could be validated in the technical validation, were further analyzed in an independent validation set (n = 82). Finally survival analysis was performed on a meningioma set, composed of 87 newly diagnosed primary meningiomas without any prior treatment.

Patients and corresponding tumors were included as part of the FORAMEN effort of the Neuro-Oncology Section of the German Society of Neurosurgery (DGNC). FORAMEN is a multi-institutional study group that conducts clinical and translational projects dealing with aggressive meningiomas. Clinical follow-up data were obtained by reviewing medical records, written correspondence with the registration office, and through telephone interviews as necessary. In addition to a subset of rare WHO°III tumors with incomplete clinical data (n = 25, 3NA tumors), only meningiomas with complete tumor resection (Simpson grade 1-3, n = 119) [8,9] were included. Here recurrence-free tumors had a follow-up of at least 36 months. According to their future relapse characteristics, tumors were assigned to the following clinico-pathological subgroups: NR = tumors without any further recurrence within the observation period of at least 36 months, R = subsequent recurrent tumor of the same WHO grade after complete resection (Simpson grade 1-3), M = subsequent recurrent tumor of a higher WHO grade after complete resection (Simpson grade 1-3), NA = no further follow-up available or incomplete resection (Simpson grade ≥4) (Figure 1, Table 1).

**Table 1:** Definition of clinical subcategories within histological WHO grades

WHO°	Clinico-pathological subgroup	Definition
WHO°I	1NR	WHO°I without any further recurrence within the observation period
	1R	WHO°I with subsequent recurrent tumor of WHO°I
	1M	WHO°I with subsequent recurrent tumor of WHO°II or WHO°III
WHO°II	2NR	WHO°II without any further recurrence within the observation period
	2R	WHO°II with subsequent recurrent tumor of WHO°II
	2M	WHO °II with subsequent recurrent tumor of WHO°III
WHO°III	3NR	WHO°III without any further recurrence within the observation period
	3R	WHO°III with subsequent recurrent tumor of WHO°III
	3NA	WHO°III with no further follow-up available

Meningiomas were categorized according to their WHO grade and their future clinical behavior: NR = non recurrent (meningioma without future recurrence), R = recurrent (meningioma with future recurrence of the same WHO grade), M = tumors with malignant progression (meningioma with future recurrence as higher WHO grade), NA = no available clinical data for classification; observation period ≥ 36 months.

To perform expression analysis and subsequently validate the data in an independent study sample, tumors were split into two separate tumor sets. The microarray study (discovery) sample consisted of 62 meningiomas (WHO°I: n = 20, WHO°II: n = 14, WHO°III: n = 28) for which tumor tissue was available in the departmental tumor banks following surgery at the Departments of Neurosurgery at the University Hospitals Heidelberg and Bonn. The validation set was composed of the remaining 82 meningioma samples (WHO°I: n = 17, WHO°II: n = 34, WHO°III: n = 31) contributed by the Departments of Neurosurgery at the University Hospitals in Heidelberg, Bonn, Homburg, Hamburg and Würzburg. WHO grading was performed by board-certified neuropathologists according to the WHO classification of 2007. Consecutively, the major criterion was mitotic count. In cases displaying clearly one of the other cytological criteria of higher grade, this criterion was applied. However, no samples with strong divergence (e.g. rhabdoid with no proliferative activity) were found. Diagnosis of the non-recurring WHO°III meningiomas (3NR) were determined by a board-certified neuropathologist of the respective contributing hospital and independently confirmed by neuropathologists of the Dpt. of Neuropathology, University Hospital Heidelberg, Germany. For clinico-pathological characteristics of both study samples see Table 2.

**Table 2:** Clinical and histological characteristics of meningiomas from microarray discovery set and independent validation set

Characteristics	Microarray set		Validation set	
	N	%	N	%
**Sex**				
Male	24	38.7	41	50
Female	38	61.3	41	50
**Age at 1st diagnosis [years]**				
Median	56.2		59.25	
Range	18-83		23-87	
**WHO Grade**				
WHO°I	20	32.2	17	20.7
WHO°II	14	22.6	34	41.5
WHO°III	28	45.2	31	37.8
**Subtype**				
Fibroblastic	6	9.7	2	2.4
Meningothelial	4	6.5	3	3.7
Transitional	9	14.5	11	13.4
Atypical	14	22.6	26	31.7
Angiomatous	0	0	0	0
Clear cell	0	0	1	1.2
Secretory	0	0	1	1.2
Anaplastic	24	38.7	17	20.7
Rhabdoid	2	3.2	0	0
Papillary	2	3.2	0	0
Unknown	1	1.6	21	25.6
**Location**				
Frontal	8	12.9	7	8.5
Convexity	23	37.1	26	31.7
Falx	6	9.7	10	12.2
Tentorial or parasagittal	15	24.2	17	20.7
Cranial base	10	16.1	14	17.1
Other	0	0	8	9.8
**Primary or recurrent tumor**				
Primary tumor	34	54.8	59	72.0
Recurrent tumor	28	45.2	22	26.8
Unknown	0	0	1	1.2
**Resection grade**				
Simpson° 1	33	53.2	-	-
Simpson° 2	12	19.4	-	-
Simpson° 1 or 2	-	-	64	78.0
Simpson° 3	11	17.7	11	13.4
Simpson° 4	6	9.7	6	7.4
Simpson° 5	0	0	1	1.2
**Postoperative treatment**				
Radiotherapy	20	32.3	19	23.2
Chemotherapy	3	4.8	2	2.4
**Clinical progression**				
Recurrence with same WHO°	23	37.1	27	32.9
Recurrence with higher WHO°	9	14.5	8	9.8
No recurrence	29	46.8	44	53.7
Unknown	2	3.2	3	3.6
3-year progression-free time**				
WHO°I	17	85	13	76.5*
WHO°II	9	64.3	18	52.9*
WHO°III	6	21.4	5	16.1*
3-year survival**				
WHO°I	18	90	15	88.2*
WHO°II	10	71.4	32	94.1*
WHO°III	10	35.7	8	25.8*
Follow-up**	Median [months]		Median [months]	
WHO°I	82.5		110	
WHO°II	103		120.5	
WHO°III	93.5		19	

* percentage of tumors of respective WHO grade

** only newly diagnosed primary meningiomas without any prior treatment

After combining the discovery and the validation sets, progression-free survival (PFS) could be studied in a subset of 87 treatment-naïve meningioma patients, who had undergone complete tumor resection (clinico-pathological characteristics Supplementary Table S1). These newly diagnosed patients had never received any prior radio- or chemotherapeutical treatment. This allowed studying potential survival associations of novel biomarkers independent of possible confounders such as the extent of resection (EOR) and treatment-related expression changes. Finally, we were able to study matched pairs of primary and recurrent tumors from a total of 13 patients.

### Tumor material, quality control and RNA extraction

Fresh tumor material obtained intraoperatively was snap-frozen and stored at −80°C until further processing. Only samples with a vital tumor cell content > 60 % as determined on H&E stained slides obtained from each tissue used by a board-certified neuropathologist were eligible (FS, Dpt Neuropathology, University Hospital Heidelberg, Germany). Furthermore Ki67 and PHH3 indices were provided by a neuropathologist. Total RNA was extracted from tissues using the AllPrep Kit (Qiagen) according to the manufacturer's instructions. RNA integrity was assessed by the Agilent 2100 Bioanalyzer.

### Microarray analysis and data normalization

1 μg total RNA from 62 meningioma tissues was submitted to the Genomics Core Facilities of the German Cancer Research Center (DKFZ, Heidelberg, Germany) for microarray analysis. After purification, reverse transcription into cDNA and labeling according to the Illumina protocol [30], samples were hybridized to Human HT-12 V.4.0 arrays (Illumina). Raw-intensity data were obtained after image analysis of the fluorescent spot intensity reads. All preprocessing and normalization steps were performed in the *R* programming environment [www.r-project.org]. Inter-array normalization was conducted using *qspline* normalization in the *affy* package [31,32]. After median probe set summarization, a linear model was fitted to account for different batches (*limma* package). Lastly, intra-array normalization was performed by means of median-centering data and followed by log2 transformation. Data were deposited at NCBI Gene Expression Omnibus (GEO accession number GSE74385).

### cDNA synthesis and quantitative PCR

To confirm expression of selected genes, real-time quantitative PCR (qPCR) analysis was conducted. 1 μg of total RNA was reverse transcribed with a Transcriptor cDNA First Strand Synthesis Kit (Roche) and random hexamer primers. qPCR was performed in triplicates on a LightCycler 480® (Roche) using the LightCycler 480® Probes Master and probes from the Universal Probe Library (Roche) as described [www.roche-applied-science.com]. Relative expression values were determined for each sample using the housekeeping genes beta-actin *(ACTB)* and guanine nucleotide binding protein 1 *(GNB1).* Primer sequences are listed in Supplementary Table S2.

### Immunohistochemistry

3μm paraffin sections were incubated and processed on a Ventana BenchMark XT® immunostainer (Ventana Medical Systems). Antibodies were anti-human PTTG1 (1:20, Life Technologies) and anti-human LEPR (1:50, Abcam). The Ventana staining procedure included pretreatment with cell conditioner 2 (pH 6) for 60 min (PTTG1) or cell conditioner 1 (pH 8) for 60 min (LEPR) followed by incubation with primary antibody at 37°C for 32 min. Incubation was followed by Ventana standard signal amplification, UltraWash, counterstaining with one drop of hematoxylin for 4 min and one drop of bluing reagent for 4 min. For visualization, ultraView™Universal DAB Detection Kit (Ventana Medical Systems) was used. LEPR and PTTG1 exhibited different staining patterns. While LEPR showed rather homogeneous staining of larger areas with slight variations over the section, PTTG1 staining was found in single cells what allowed for exact counting of the positive cells. This pattern also allowed for evaluation of LEPR over the entire section while the heterogeneity of PTTG1 was analyzed in representative fields. Noteworthy, the feasibility of practical application of this marker would be limited if the entire section needed to be evaluated for single positive cells. Staining pattern was evaluated applying a modified H-Score [33] for LEPR. The score ranges from 0 to 300 and is calculated as the percentage of weakly stained cells plus the percentage of moderately stained cells multiplied by two plus the percentage of strongly stained cells multiplied by three. For PTTG1, number of positive cells was counted in the high-power-field (40x) with the highest density of positive cells. In order to compare the predicting capability of our markers with established markers in meningioma diagnosis, the expression of established proliferation markers Ki67 (MIB-1 antibody) and PHH3 were assessed and scored as described [34].

### Statistical analysis

Unless otherwise stated, statistical analyses were conducted in *R* [www.r-project.org]. Differential expression in meningioma subgroups was assessed using Student's t-test. False discovery rates (FDRs) were estimated using a permutation based approach [35]. For survival analysis, PFS was used as an end point. Prognostic significance was determined using univariate and multivariate Cox regression analysis and log-rank tests. For multivariate models, all clinico-pathological parameters significant in univariate analysis were included.

### Ethical approval

Local ethical committees approved the study. Written informed consent for translational research was obtained from all patients.

## RESULTS

### Characterization of meningioma study samples

A 62-patient discovery and an 82-patient validation set were collected from five German university hospitals. In total, they comprised 59 WHO°III, 48 WHO°II and 37 WHO°I meningiomas. Of note, a high number of recurrent WHO grade I and II meningiomas with and without malignant progression (n = 39; 1M+R and 2M+R) were included. To account for known prognostic markers, we analyzed Ki67 and PHH3 expression. As expected, both markers showed differential expression between the WHO grades, but did not predict clinical behavior within a given WHO grade as represented by our clinical subgroups (Supplementary Figure S1).

### Aggressive meningiomas share a transcriptional profile across different WHO grades

To characterize the transcriptional landscape of aggressive meningiomas, we assessed the global transcriptional differences in our discovery set of 62 tumors (Figure 1) using microarray analyses and an unsupervised approach. Employing principal component analysis (PCA), we observed a spectrum of transcriptional profiles lining up according to the WHO grade as well as to the clinical subgroup (i.e. NR, non-recurrent; R, recurrent without malignant progression; M, malignant progression) in principal component 1 (PC1) (Supplementary Figure S2). In contrast, WHO°I NR (1NR) and WHO°III tumors marked the extreme ends, WHO°II tumors, as well as the more aggressive WHO°I M+R (1M+R) tumors were located in between.

**Figure 2:**
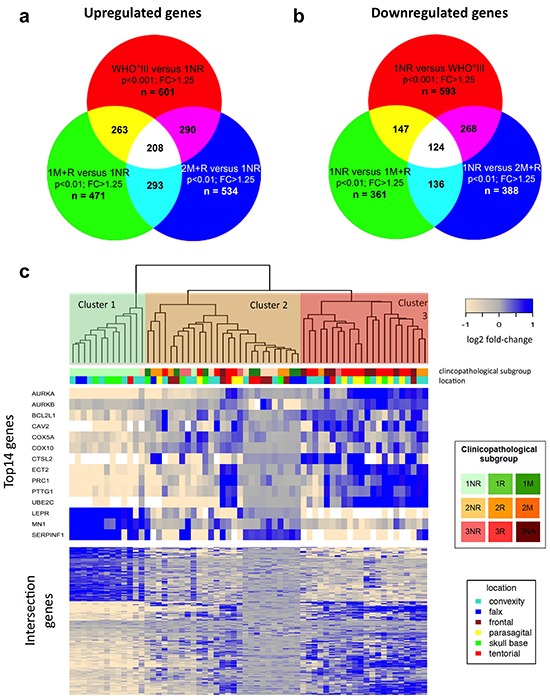
Comparative transcriptomics in meningiomas **a.** Intersection study of upregulated genes in recurrent and malignant meningiomas. Venn diagram showing overlap of overexpressed genes in 1M+R, 2M+R and WHO°III as compared to 1NR tumors. Cut off: p < 0.01 (for WHO°III p < 0.001) and fold change (FC) > 1.25. **b.** Intersection study of upregulated genes in non-recurrent WHO°I meningiomas. Venn diagram showing overlap of overexpressed genes in 1NR tumors compared to 1M+R, 2M+R and WHO°III tumors. Cut off: p < 0.01 (for WHO°III p < 0.001) and fold change (FC) > 1.25. **c.** Heatmap of gene expression malignancy signature for relapsing, malignant progressing and WHO grade III meningiomas. Clustering of microarray set, using gene malignancy signature (n = 332) generated with intersection studies.

Next, we investigated the extent of transcriptional differences between each of our tumor subgroups and combinations thereof by applying comparative statistics. Estimating the false discovery rate (FDR) revealed robust differences between the following clinical subsets: 1NR vs 1M+R; 1NR vs 2M+R; 1NR vs WHO°III, 2NR vs WHO°III (Supplementary Table S3). Accordingly, we performed intersection studies between 1NR tumors and aggressive (recurrent, malignantly progressing or WHO grade III) meningiomas (1M+R, 2M+R and WHO°III). For this purpose, we generated lists of top differentially expressed genes (p < 0.01; fold change > 1.25) for the respective groupings (Figure 2A, 2B). In order to obtain a comparable number of differentially expressed genes for an intersection study a stricter p-value (p < 0.001) was chosen for WHO°III vs 1NR. Interestingly, differential expression of a large set of genes (n = 332; Supplementary Table S4) was shared between aggressive meningioma subgroups across different WHO grades. 208 genes were found to be upregulated and 124 genes downregulated in the more aggressive tumor subgroups. Further pathway enrichment and gene function analyses of the top differentially expressed genes revealed enrichment for genes involved in mitosis and cell cycle in all three clinical subgroups with poor outcome (1M+R, 2M+R, WHO°III) when compared to 1NR tumors (Supplementary Figure S3–S8).

Using this malignancy signature of 332 genes to cluster our microarray study sample, we identified three major clusters (Figure 2C lower part of heatmap). Cluster 1 almost exclusively contained 1NR tumors, while cluster 2 was enriched for WHO°II tumors, recurrent WHO°I tumors (1R + 1M) and non-progressing WHO°III tumors (3NR). In contrast, in cluster 3 we primarily found 3R tumors. We selected 13 top candidate genes for further validation based on strong differential expression (WHO°III vs I: p < 0.01; FC > quintile 90 or FC < quintile 10 and in at least two clinico-pathological subgroup comparisons p < 0.01; FC > 1.25) and cancer-associated functional properties (Figure 2C, upper part of heatmap). Based on its increasing importance as a cancer-related, druggable molecule [36–40] and its differential expression between WHO°III and °I (p < 0.01), we additionally included AURKB in our analysis.

### Aggressiveness of meningiomas is associated with upregulation of *PTTG1, AURKA, AURKB, ECT2, PRC1, UBE2C, COX5A* and *COX10* and downregulation of *LEPR* and *MN1*

**Figure 3:**
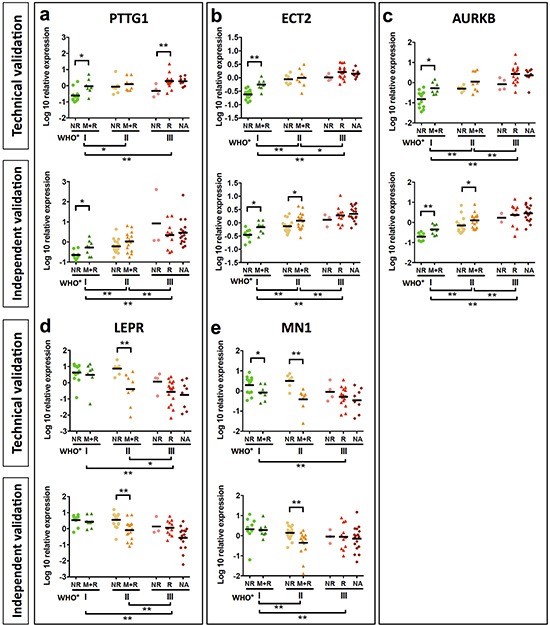
Validation of candidate genes by qPCR Analysis of *PTTG1* **a.** *ECT2* **b.** *AURKB* **c.** *LEPR* **d.** and *MN1* **e.** mRNA expression of candidate genes was analyzed in samples of the discovery set (a-e upper graph). A further validation was performed in an independent meningioma set (a-e lower graph). Mean mRNA expression of *PTTG1, ECT2* and *AURKB* shows a significant increase. Statistical significance was determined by Mann-Whitney test, *p < 0.05, **p < 0.01.

Next we analyzed expression of our 14 candidate genes by qPCR. For 10 of these genes, differential expression between clinico-pathological subgroups was confirmed in the initial discovery set. Among these, 8 genes were upregulated and 2 genes downregulated in recurrent, malignantly progressing and WHO grade III meningiomas (Supplementary Table S5; Figure 3; Supplementary Figure S9). In order to challenge the diagnostic capability of these 10 genes, we next went for validation in an independent study sample comprising 82 meningiomas WHO°I to °III (validation set Figure 1, for clinico-pathological information see Table 2). Significant discrimination between WHO grades was confirmed for all 10 genes analyzed. In addition, *PTTG1, AURKB, ECT2, PRC1* and *UBE2C* expression successfully distinguished between 1NR and the more aggressive 1M+R tumors, and the *AURKB, ECT2, MN1, LEPR* gene expression discriminated between 2NR and 2M+R tumors (Figure 3, Supplementary Figure S9). However, the low number of non-recurring WHO°III tumors in both study samples (3NR) did not allow for a robust discrimination between 3NR and 3R subgroups although a similar trend was clearly visible for some of the genes in the initial discovery set as well as in the independent validation set.

To exclude that these findings are influenced by tumor treatment, we repeated our analysis for 87 newly diagnosed meningiomas extracted from both study samples (n = 87). None of these patients received any tumor treatment prior to surgery except for corticosteroids in cases of severe edema. Noteworthy, the significant transcriptional differences between all WHO grades were confirmed for each candidate gene. Furthermore, *PTTG1, AURKA, AURKB, ECT2, PRC1, UBE2C, COX5A* and *COX10* expression was able to discriminate between 1NR and 1M+R tumors and *LEPR* and *MN1* between 2NR and 2M+R tumors (Supplementary Figure S10–S12).

**Figure 4:**
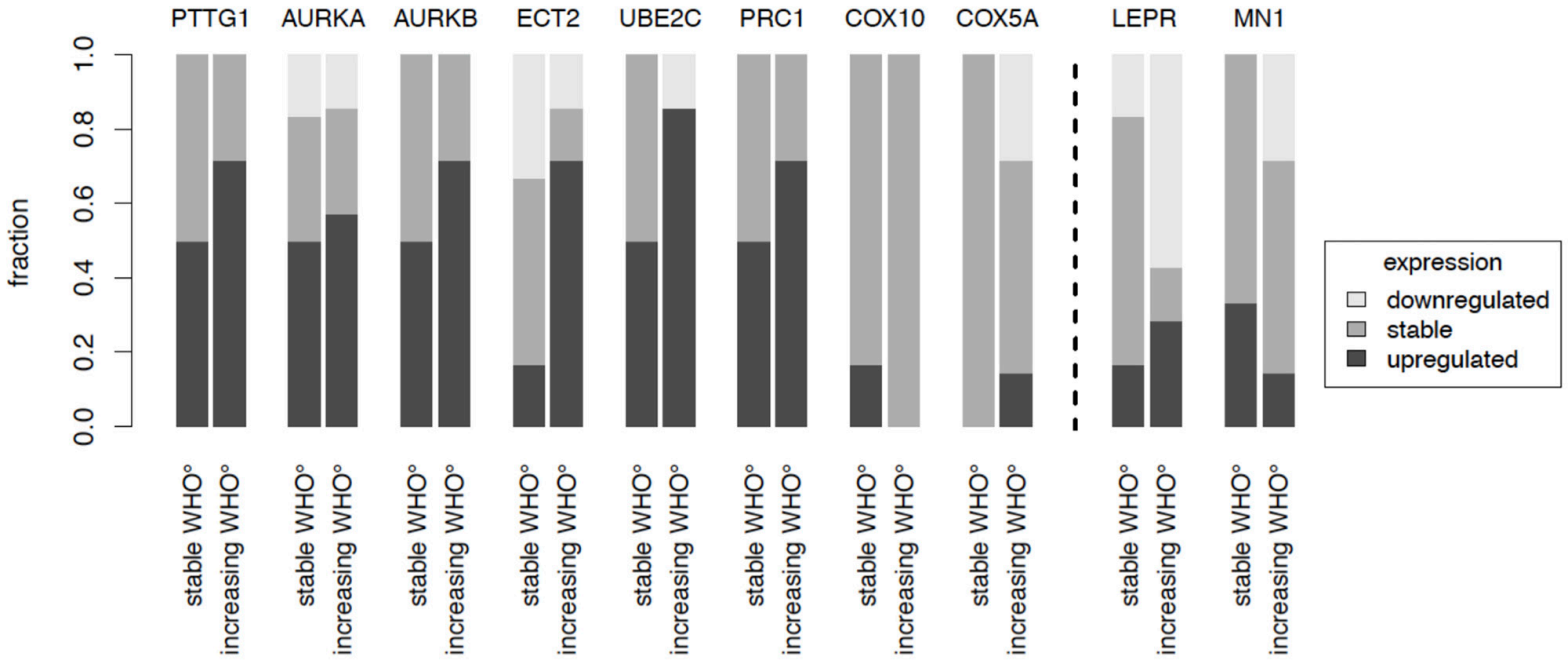
Recurrence patterns The gene expression of candidate genes in pairs of recurrent tumors of the same patient was analyzed. Expression changes were studied in patients where WHO grade did not change (n = 6) as compared to patients where recurrent tumors showed an increased WHO grade (n = 7). Increase was defined as ≥ 2 fold change, decrease as ≤ 0.5 fold change.

To further investigate the association of our candidate genes with tumor progression, we analyzed gene expression in tumor samples of the same patient obtained at different surgical time points (Figure 4). This included 6 tumor pairs from patients without changes in WHO grade („stable WHO grade“) and 7 tumor pairs from patients undergoing malignant progression („increasing WHO grade“). Increasing gene expression was defined as ≥ 2 fold change and decreasing gene expression as ≤ 0.5 fold change. Although more pronounced in some of the candidates, expression of genes related to a shorter PFS *(PTTG1, AURKA, AURKB, ECT2, UBE2C, PRC1, COX5A, COX10)* almost never decreased in recurrent tumors. Instead they were often found at higher expression levels in recurrent tumors with malignant progression as compared to recurrent tumors with stable WHO grade. For genes associated with a prolonged PFS (*LEPR*, *MN1*), we observed the opposite phenomenon (Figure 4). These findings further suggest that our candidate genes play a critical role in tumor progression through up- or downregulation in the recurrence and might represent potential therapeutic targets for recurrent meningiomas.

### Expression of *PTTG1* and *LEPR* predicts survival independent of WHO grade, extent of resection and gender

So far, our experiments had successfully confirmed an association between differential expression of 10 candidate genes with tumor recurrence and WHO grade in two independent tumor sets. Hence, we finally sought to investigate expression of our candidate biomarkers as prognostic factors against a clinical background. To this end, the impact of differential expression of our candidate genes on PFS was assessed in 87 patients with treatment-naïve, newly diagnosed meningiomas undergoing complete surgical resection (Simpson grade 1–3). This allowed for studying survival associations of our novel markers independent of possible confounders such as EOR and treatment-related expression changes due to ionizing radiation or cytotoxic agents.

**Figure 5:**
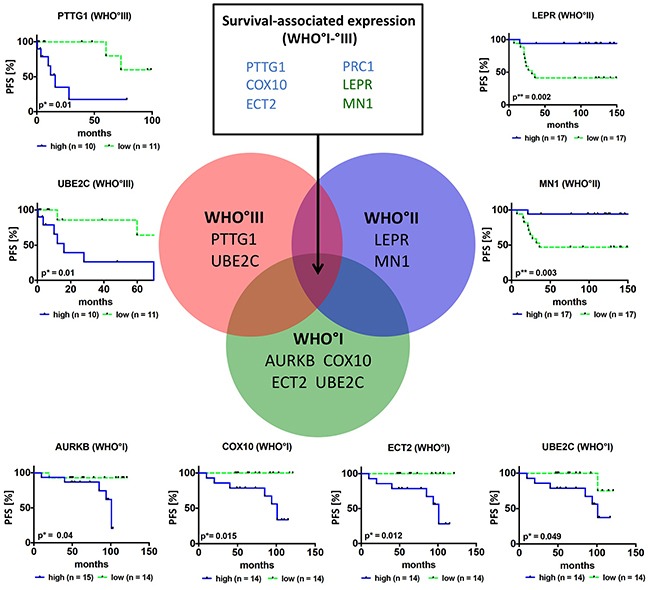
Survival association of candidate genes in primary meningiomas Venn diagram and Kaplan-Meier plots show candidate genes, where expression is associated with PFS in the whole study sample (n = 6, list of genes in upper middle rectangle, blue = higher expression results in worse survival, green = higher expression results in improved survival) or is associated with PFS in a specific grade. For survival analysis only newly diagnosed and complete resected (Simpson grade 1–3) primary tumors (n = 87) were included. Patients were categorized into two groups according to their median mRNA expression levels into high (blue curve) and low (green curve) expression *p < 0.05, **p < 0.01.

Univariate analyses revealed that 8 of the 10 candidate genes (*PTTG1, AURKB, ECT2, PRC1, COX10, MN1, LEPR* and *UBE2C,* Figure 5; Supplementary Figure S13-S20) indeed were associated with PFS: Higher expression of *PTTG1, AURKB, ECT2, PRC1* and *COX10* as well as lower expression of *LEPR* and *MN1* resulted in a significantly shorter PFS. In order to compare gene expression-associated survival independent of WHO grade, PFS was further analyzed in meningioma subsets of the same WHO grade. Here higher *AURKB, ECT2, COX10* and *UBE2C* expression was associated with worse PFS in WHO°I tumors (Figure 5). In WHO°II meningiomas reduced expression of *LEPR* and *MN1* resulted in a significantly shorter PFS, while in WHO°III meningiomas this was the case for a higher expression levels of *PTTG1* and *UBE2C* (Figure 5). Of all clinical parameters tested, male gender (HR = 2.86; p = 0.005) and WHO grade (WHO°III vs °I, HR = 3.42; p = 0.0153) were significantly associated with shorter PFS (Table 3A).

**Table 3:** Survival analysis in newly diagnozed, primary meningioma cases (n = 87)

**(a) Univariate analysis of clinical confounders.**			
	**HR**	**95%-CI**	**p-value**
**age (continous)**	1.028	0.996-1.061	0.090
**male**	2.866	1.327-6.190	0.005**
**WHO° II vs I**	1.288	0.496-3.342	0.603
**WHO° III vs I**	3.420	1.265-9.241	0.015*
**Simpson grade II vs I**	1.054	0.404-2.751	0.915
**Simpson grade III vs I**	2.282	0.916-5.687	0.077
**radiotherapy**	1.352	0.567-3.223	0.495
Results of Cox proportional hazard analysis are summarized. P-values were calculated employing log-rank test (*p < 0.05, **p < 0.01). Age was included into the model as a continuous variable. WHO and Simpson grades were used as categorical variables.			
All clinico-pathological confounders significant in the univariate analysis were included in the multivariate model. Results of Cox proportional hazard analysis are summarized. P-values were calculated employing log-rank test (*p < 0.05). WHO grades were used as categorical variables. HR = hazard ratio. 95%-CI = lower and upper border of 95% confidence interval.			
**(b) Multiple survival analysis.**			
	**HR**	**95%-CI**	**p-value**
**WHO° II vs I**	0.905	0.342-2.396	0.841
**WHO° III vs I**	1.190	0.402-3.527	0.753
**Male**	2.325	1.056-5.121	0.036*
**PTTG1**	2.490	1.077-5.759	0.033*
**LEPR**	0.286	0.109-0.748	0.011*

All clinico-pathological confounders significant in the univariate analysis were included in the multivariate model. Results of Cox proportional hazard analysis are summarized. P-values were calculated employing log-rank test (*p < 0.05). WHO grades were used as categorical variables. HR = hazard ratio. 95%-CI = lower and upper border of 95% confidence interval.

**Figure 6:**
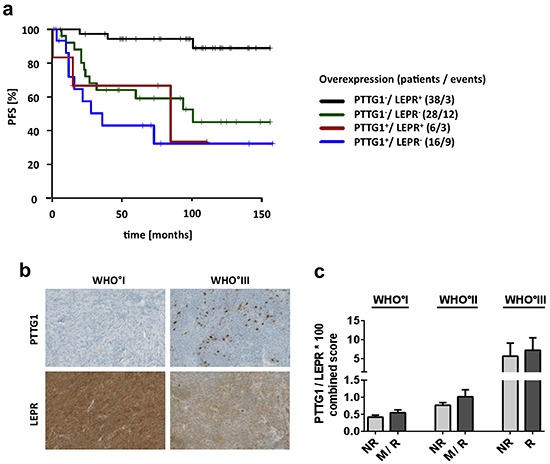
Survival association and protein expression of PTTG1 and LEPR **a.** Survival prediction of combined *PTTG1* and *LEPR* mRNA expression. Patients were divided into four groups. *PTTG1* expression was dichotomized by highest quartile of expression and *LEPR* by median expression of all samples. + = group with high expression. **b.** Immunohistochemical staining of PTTG1 and LEPR. Pictures show representative samples of increased numbers of PTTG1-positive cells and reduced expression of LEPR in WHO grade III meningiomas. **c.** Staining results of 36 tumors represented by a combined score of PTTG1 and LEPR (PTGG1 × 100/LEPR) assigned to clinico-pathological subgroups. Each subgroup contains a minimum of 4 up to a maximum of 8 tumors. Error bars = SEM, NR = non-recurrent, R = recurrent, M = tumors with malignant progression.

To enable a meaningful multivariate analysis in this medium-sized study sample, we only assessed the two genes with the lowest multiplicity-unadjusted p-value (< 0.001), *PTTG1* and *LEPR,* in a model adjusting for WHO grade and gender. In this multivariate model, both *PTTG1* and *LEPR* expression were found to be independent predictors of patient PFS (Table 3B). However, the patient group with the longest PFS was characterized by co-occurrence of low *PTTG1* and high *LEPR* mRNA expression (Figure 6A). Finally, immunohistochemical staining of these two genes confirmed that the observed expression changes translate onto protein level (Figure 6B, Supplementary Figure S21) and that combined staining data from both genes to a common score show a close association with malignant WHO grade III meningiomas and thus the aggressiveness of meningiomas (Figure 6C).

## DISCUSSION

In search for new biomarkers and putative drug targets of clinically aggressive meningiomas, we performed a comprehensive analysis of the transcriptomic landscape in a large, multicenter study sample consisting of 144 meningiomas WHO°I to °III. The comparably high number of WHO°II and °III meningiomas as well as the assignment of tumors according to their future clinical behavior into non-recurrent (NR) and recurrent (R) meningiomas and meningiomas undergoing malignant progression (M) allowed for the identification of a WHO grade-independent 332 gene signature shared by all clinically unfavorable meningioma subgroups. Survival association was confirmed for 8 (*PTTG1, AURKB, ECT2, COX10, PRC1, UBE2C, LEPR* and *MN1*) of these genes in two independent study samples as well as in matched pairs of primary and recurrent tumors of the same patient. Moreover, multivariate survival analysis established increased PTTG1 and decreased LEPR expression as novel and potentially powerful prognostic markers for the identification of clinically aggressive meningiomas.

To date, the prognostic assessment of intrinsic aggressiveness of meningiomas is based on the histological WHO classification sometimes supported by proliferation markers such as Ki67 and PHH3 [4, 5, 41, 42]. However, this approach often fails to predict the prognosis of individual meningioma patients so that histological classification frequently differs from the future clinical aggressiveness [4, 5, 43, 44]. In other cancer entities gene expression profiling has been successfully applied to identify diagnostic markers and therapeutic targets and also to refine disease classification [11, 45–48]. In breast cancer, first trials were launched to prospectively validate the performance of a new prognostic RNA-based tool (70-gene profiler MammaPrint™) [49]. Attempts to associate gene expression patterns with malignancy in meningioma have been made [23, 24, 26, 28, 44, 50–53], but in most cases did not surpass comparison between the routinely assessed WHO grades or included low numbers of malignant WHO grade III meningiomas. In a meta-analysis, Stuart et al. compared gene expression profiles of WHO°I and WHO°III meningiomas across multiple sets and could validate 11 differentially expressed genes via qPCR [28]. Pérez-Magán et al. identified a 49–gene signature associated with tumor progression and malignancy in meningiomas based on meta-analysis of data from five different microarray platforms [24]. Although our study included only one independent validation set, we were the first to use a uniform microarray platform for all tumors and still included a greater number of less frequent WHO°II and rare °III tumors. Corroborating our approach, unsupervised analyses indeed revealed that transcriptional differences do not only exist between different WHO grades, but even between non-recurrent (1NR) and clinically more aggressive (1R and 1M) WHO°I meningiomas. Subsequent intersection studies between our distinct clinico-pathological subgroups further support the existence of intrinsic transcriptional differences between the clinically benign WHO°I NR meningiomas and clinically more aggressive meningiomas (1M+R, 2M+R, WHO°III) by identifying commonly up- and downregulated genes. Of note, 34 % of top downregulated genes (n = 124) and even 44 % of top upregulated genes (n = 208) are shared by all clinically aggressive meningioma subgroups across the different WHO grades and can be merged to a novel 332-gene signature indicating biological aggressiveness.

These findings further suggest that some transcriptional changes associated with a worse clinical outcome already occur quite early in more aggressive R+M WHO°I meningiomas, rather than being a gradually gained effect of clonal evolution from WHO°I to WHO°II/°III tumors.

Considering that a malignancy signature of 332 genes might be difficult to apply in clinical practice, we challenged the prognostic potential of 13 of these genes, which were selected based on their strong expression differences, pathway analysis and literature research. Indeed, the heatmap in Figure 2C illustrates a differential expression of these candidate genes between three malignancy-related tumor clusters. Moreover, subsequent qPCR (Figure 3, Supplementary Table S5, Supplementary Figure S9) not only confirmed their capability to discriminate between WHO grades but for most of the genes to predict aggressive clinical behavior especially in WHO°I tumors. This observation corroborates the robustness of our approach in identifying disease-relevant genes not only in late-stage tumors. For 10 of the most promising genes the large size of our study sample allowed to successfully confirm expression changes in an independent validation set as well as in matched tumor pairs from the same patient obtained at different time points of the disease. Finally, survival analysis including therapy-naïve, completely resected meningioma patients only revealed that upregulated expression of 5 of these genes (*PTTG1, UBE2C, COX10, ECT2,* and *PRC1*) as well as downregulation of *LEPR* and *MN1* was associated with a significantly reduced progression-free survival time. Even for the therapy-naïve study cohort, differential gene expression was a significant prognosticator within tumor sets defined by their WHO grades (WHO°I: *AURKB, COX10, ECT2, UBE2C*; WHO°II: *LEPR, MN1*; WHO°III: *PTTG1, UBE2C*). Most importantly, prognostic power of upregulated *PTTG1* and downregulated *LEPR* expression was confirmed to be independent of WHO grade, gender and EOR. The prognostic performance was even more pronounced when applying a combined expression score of both genes. As an important step to make use of our findings, we were able to show transcriptional changes on the protein level between WHO grade I and malignant WHO grade III meningiomas.

Given the opposite expression patterns observed for *LEPR* and *PTTG1*, diverging functional roles can be assumed. As for the leptin receptor LEPR, a loss of function in obese (fa/fa) Zucker rats is associated with increased leptin levels in the inguinal adipose tissue [54]. Since leptin resistance mainly occurs in obesity [55,56], it is worth mentioning that some studies found obesity to be significantly increased in meningioma patients [57–59]. In a microarray study of 23 meningiomas (10 WHO°I, 10 WHO°II, 3 WHO°III) and a validation set of 65 meningiomas (41 WHO°I, 24 WHO° II or III) the prognostic value of a CKS2/LEPR index in meningiomas was assumed [27]. Our study confirms the prognostic value of LEPR, even as independent predictive marker from WHO grade or EOR. However, molecular mechanisms of LEPR regulation and its influence on intracellular signaling in meningiomas are still unknown. With regard to the pituitary tumor transforming gene PTTG1 a functional role as an oncogene by regulating cell-cycle progression [60], apoptosis [61], cellular transformation [62] and the tumor microenvironment in terms of increasing the expression of proangiogenic factors [63,64] has been well established. Up to date, one descriptive study showed a PTTG1 protein expression in most meningioma tissues and an expression variability among different subtypes without analysis of clinical prognosis [65]. Moreover in a couple of cancer entities other than meningioma PTTG1 has been shown to be involved in tumor progression and to be correlated with poor prognosis [66–71].

To the best of our knowledge, one of eight disease- and survival-relevant genes identified (COX10) has never been described before to be cancer-related, while for three more genes (PTTG1, AURKB, and ECT2), a role in tumor progression and survival could only be observed in other tumor entities (Supplementary Table S6) [71–77]. In accordance with our findings, the remaining four top genes were previously found to be either upregulated (*UBE2C* and *PRC1*) or downregulated (*LEPR* and *MN1*) in WHO grade II and III meningiomas [23,27,78,79]. Moreover, our data further support the importance of these genes by identifying their differential expression even within tumors of the same WHO grade depending on their future aggressiveness.

Treatment for recurrent tumors and WHO°III meningiomas has not substantially advanced beyond surgical resection and adjuvant radiotherapy [7,80]. So far no chemotherapy has been approved as standard therapy [80]. Median survival for patients with WHO°III meningiomas of 1.5 years and the lack of a second-line therapy for recurrent meningiomas highlights the importance of detecting potential targets for new therapies [5]. The disease-relevant genes we identified might offer opportunities to develop therapeutic targets once their biological relevance and involvement in oncobiological pathways in these aggressive meningiomas of all WHO grades are more closely defined. For example, inhibitors such as Alisertib targeting one of our top genes, aurora kinase A, have already been tested in several phase I and II trials even in combination with other drugs. These studies have shown promising results in advanced solid tumors and lymphomas [36–40]. For refractory peripheral T-cell lymphomas the effect of Alisertib is currently tested in a phase III trial in combination with Pralatrexate, Gemcitabine or Romidepsin [40]. Therefore, future functional *in vitro* and *in vivo* studies of our most promising malignancy-associated genes in meningiomas including aurora kinases are warranted to further explore their therapeutical potential for the treatment of aggressive meningioma.

In summary, applying a comprehensive transcriptomic analysis in one of the largest, clinically well-characterized study samples of aggressive meningioma including primary and recurrent tumors from the same patient, we were able to identify *PTTG1, AURKB, LEPR, COX10, ECT2, PRC1, UBE2C* and *MN1* expression as potentially important prognostic markers. Four of these genes have not been associated with meningioma biology so far. The prognostic capability of *PTTG1* and *LEPR* expression was even independent of WHO grade and EOR and might therefore serve as powerful tool to select high-risk patients, who might benefit from a more aggressive therapy and closer follow-up imaging controls. Lastly, our expression data comprise a valuable *in silico* resource for further studies investigating this group of therapeutically challenging meningiomas.

## SUPPLEMENTARY FIGURES AND TABLES






