# Supplementary material for: Transcriptomic analysis of aggressive meningiomas identifies PTTG1 and LEPR as prognostic biomarkers independent of WHO grade
Source: Oncotarget. 2016 Feb 15;7(12):14551–68. doi: 10.18632/oncotarget.7396 (PMC4924735; doi:10.18632/oncotarget.7396)
Supplement: Supplementary file 1 [file oncotarget-07-14551-s001.pdf]

SUPPLEMENTARY FIGURES AND TABLES

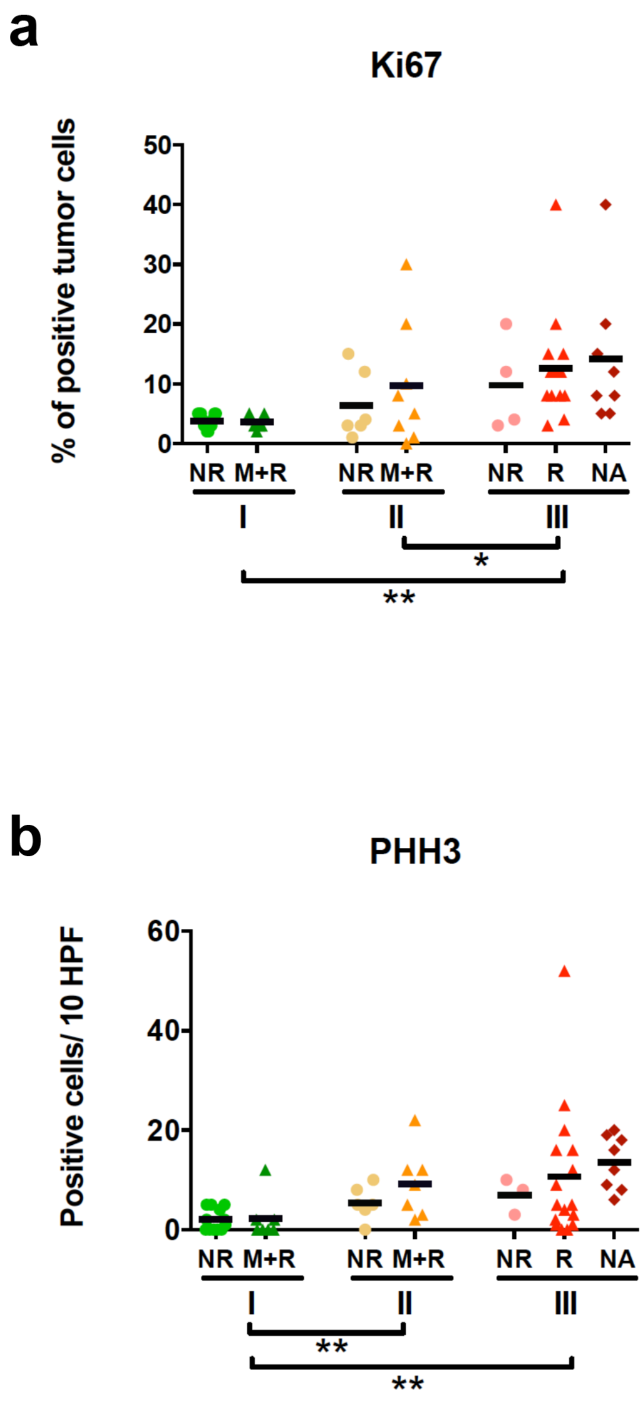

Supplementary Figure S1: Characterization of study sample with established meningioma markers a. Ki67 **b.** PHH3. Immunohistochemical stainings were performed on microarray training set. Histopathological diagnosis Ki67 and PHH3 indices were confirmed by board certified pathologists at the Department of Neuropathology (University Hospital Heidelberg, Germany). Error bars represent mean of experiments done in triplicates. Statistical significance was determined by Mann-Whitney test, *$p < 0.05$, **$p < 0.01$**.** NR = non-recurrent, R = recurrent, M = malignant progression, NA = no available clinical data for classification, HPF = high power field.

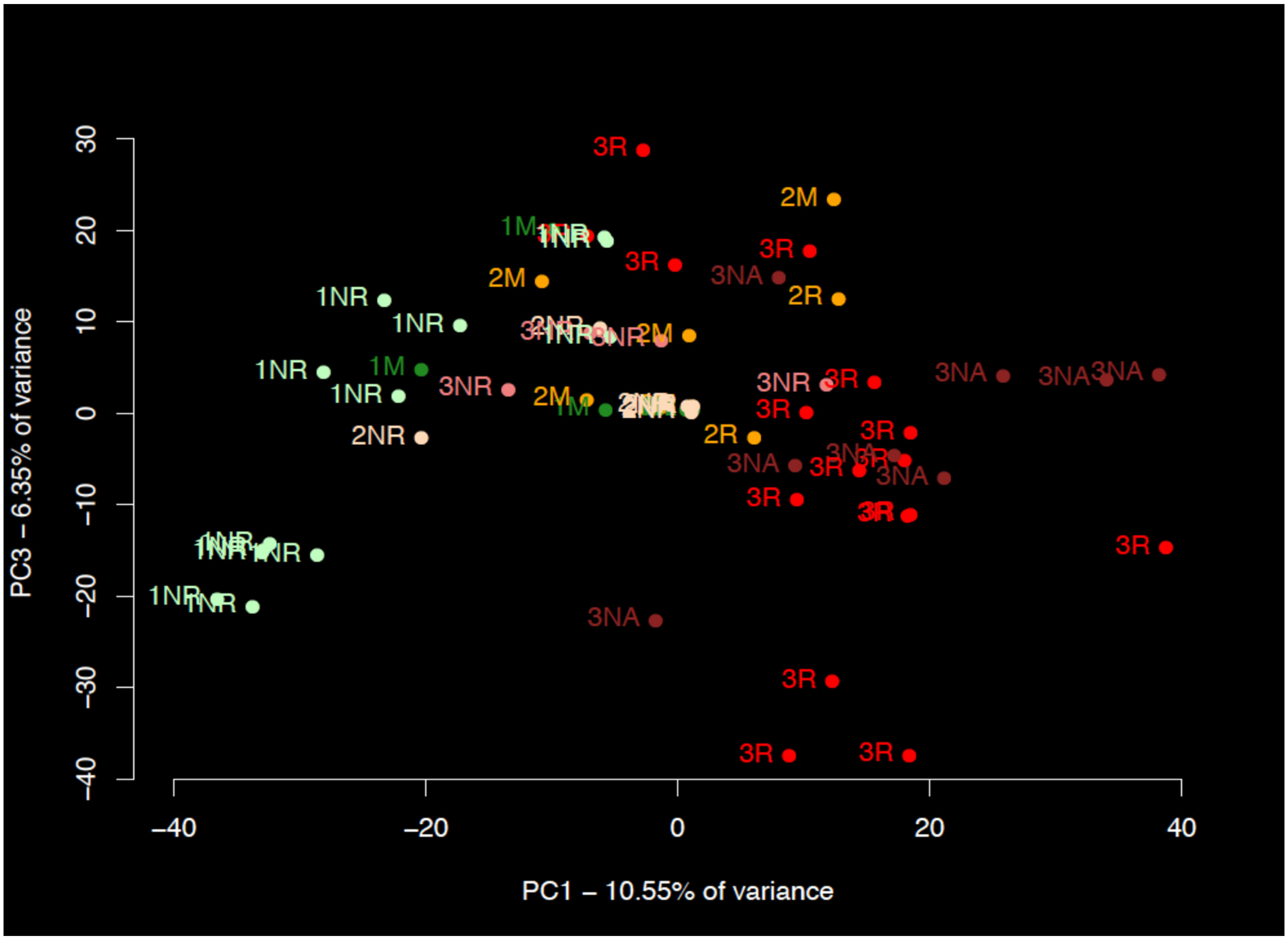

Supplementary Figure S2: Principal component 1 and 3 of principal component analysis (PCA). PCA shows global transcriptional differences in our microarray discovery set using an unsupervised approach. A marked transcriptional difference in principal component 1 between 1NR and WHO°III tumors can be observed. WHO°II tumors and 1M+R tumors are located in between. PC = principal component.

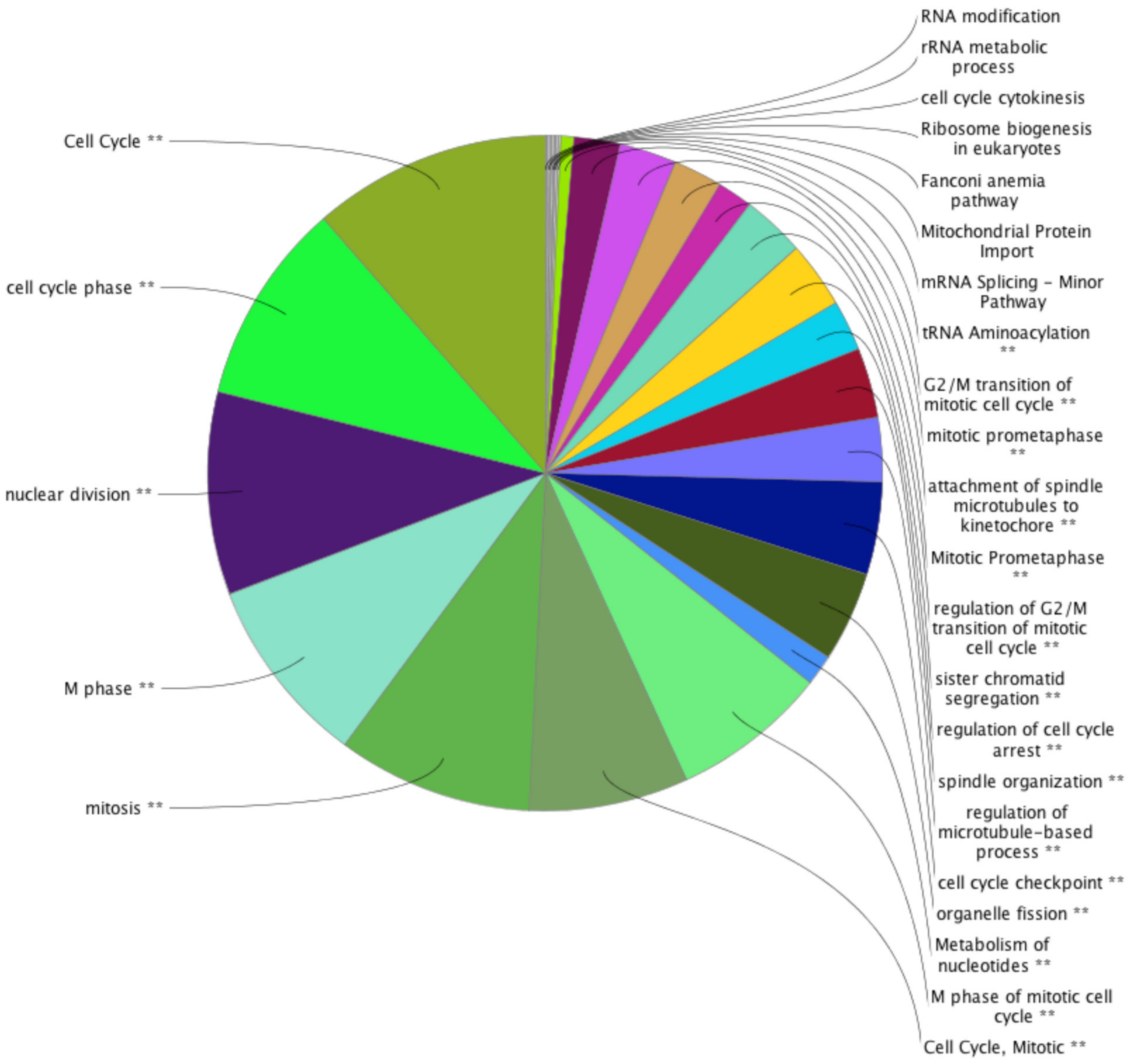

Supplementary Figure S3: Analysis of enriched pathways and gene functions in WHO°III compared to 1NR meningiomas. Analysis based on GO terms, KEGG and Reactome pathways. Charts generated by ClueGo (application of Cytoscape). Upregulated gene with $p < 0.001$ and FC > 1.25 were included in this study (n = 601).

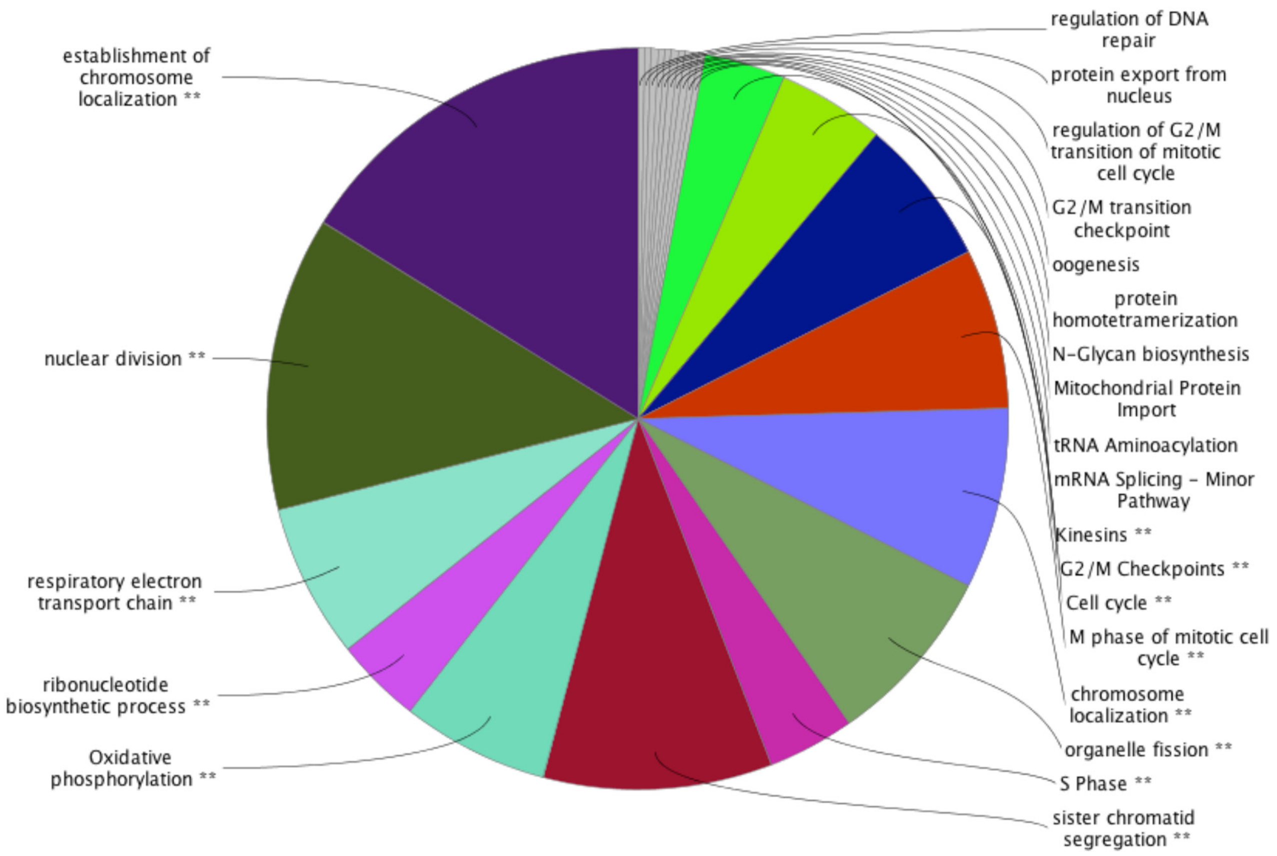

Supplementary Figure S4: Analysis of enriched pathways and gene functions in 2M+R compared to 1NR meningiomas. Analysis based on GO terms, KEGG and Reactome pathways. Charts generated by ClueGo (application of Cytoscape). Upregulated gene with $p < 0.01$ and FC > 1.25 were included in this study (n = 534).

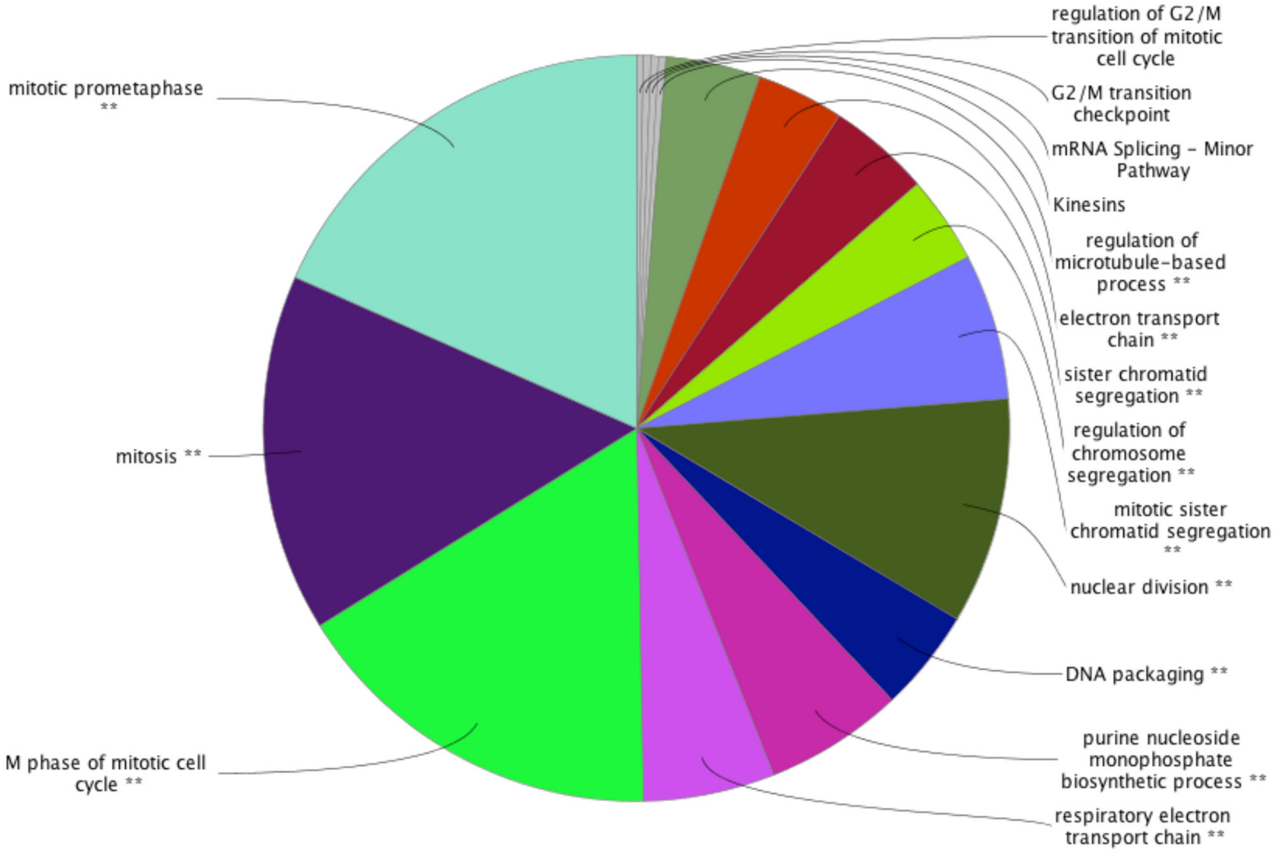

Supplementary Figure S5: Analysis of enriched pathways and gene functions in 1M+R compared to 1NR meningiomas. Analysis based on GO terms, KEGG and Reactome pathways. Charts generated by ClueGo (application of Cytoscape). Upregulated gene with $p < 0.01$ and $FC > 1.25$ were included in this study ($n = 471$).

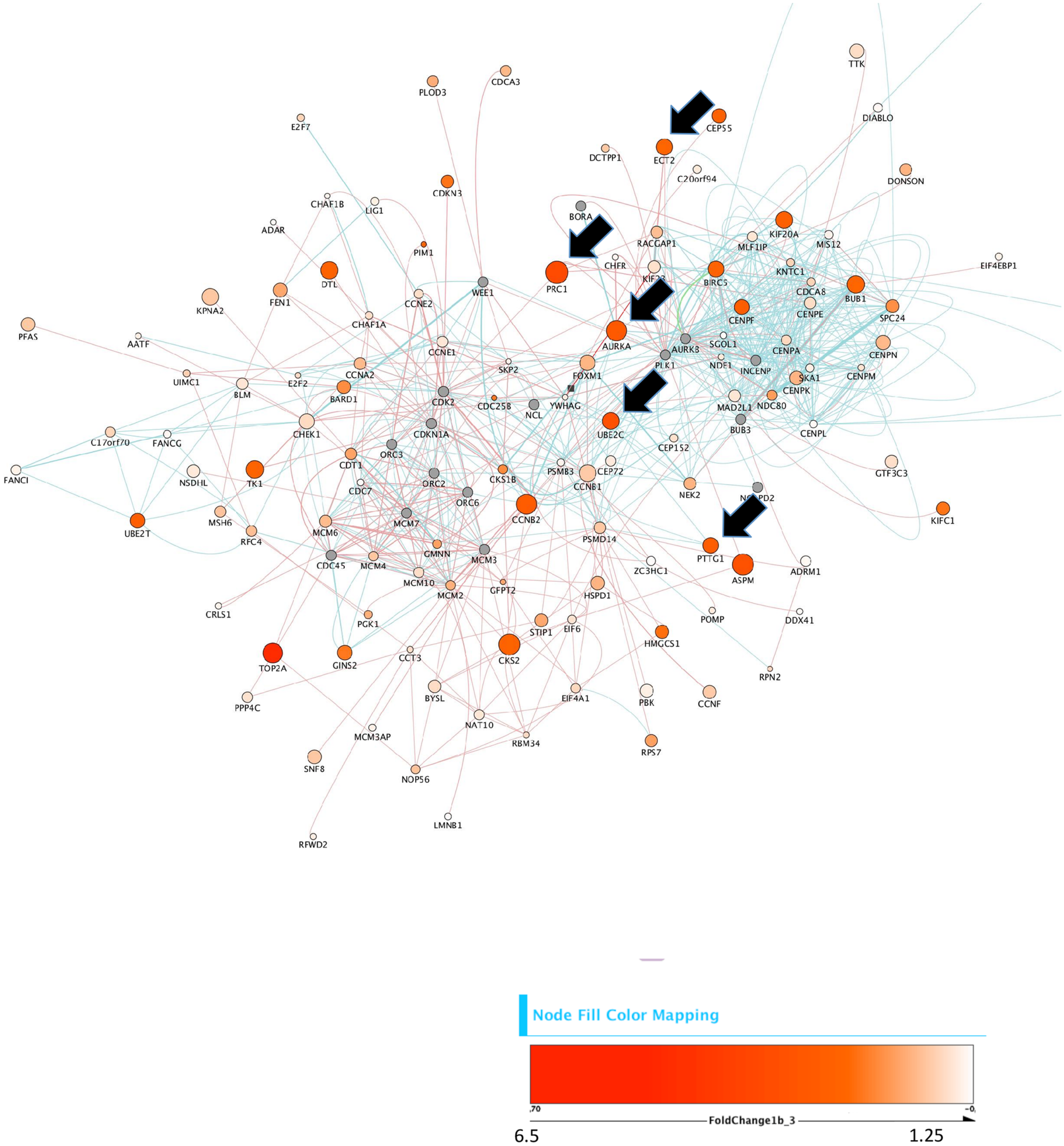

Supplementary Figure S6: Network with upregulated genes in WHO°III meningiomas involved in the cell cycle checkpoint. The network shows genes involved in the cell cycle checkpoint with their interaction partners, which are upregulated in WHO°III meningioma compared to 1NR tumors. The network was generated by Genemania (http://www.genemania.org). Green Lines = genetic interactions, blue lines = pathways, red lines = physical interactions. Upregulated genes in WHO°III with $p < 0.001$ and a FC > 1.25 were used. The node color represents the fold change between WHO°III and 1NR. The node size in the graph anticorrelates with the p-value. Arrows = genes, which were chosen for qPCR validation. WHO°III meningiomas seem to underlie a dysregulation of the cell cycle checkpoint.

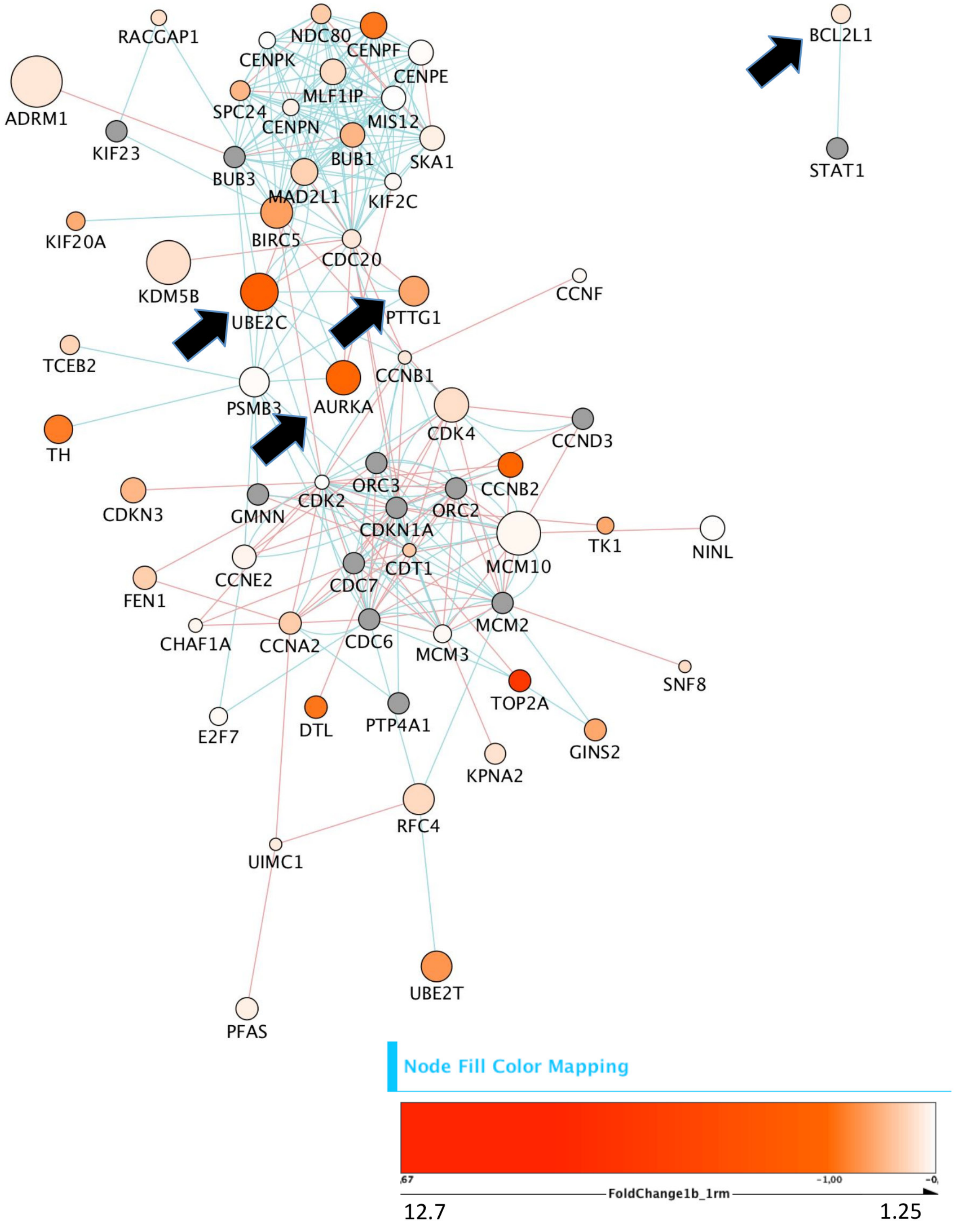

Supplementary Figure S7: Network of upregulated genes in 1M+R meningiomas involved in the cell cycle checkpoint. The network shows genes involved in the cell cycle checkpoint with their interaction partners, which are upregulated in 1M+R meningiomas compared to 1NR tumors. The network was generated by Genemania (http://www.genemania.org). Green Lines = genetic interactions, blue lines = pathways, red lines = physical interactions. Upregulated genes in WHO°III with $p < 0.001$ and a FC > 1.25 were used. The node color represents the fold change between WHO°III and 1NR tumors. The node size in the graph anticorrelates with the p-value. Arrows = genes, which were chosen for qPCR validation. 1M+R meningiomas seem to have a dysregulation of the cell cycle checkpoint, with the upregulation of similar genes as WHO°III tumors, contributing to an increased malignancy.

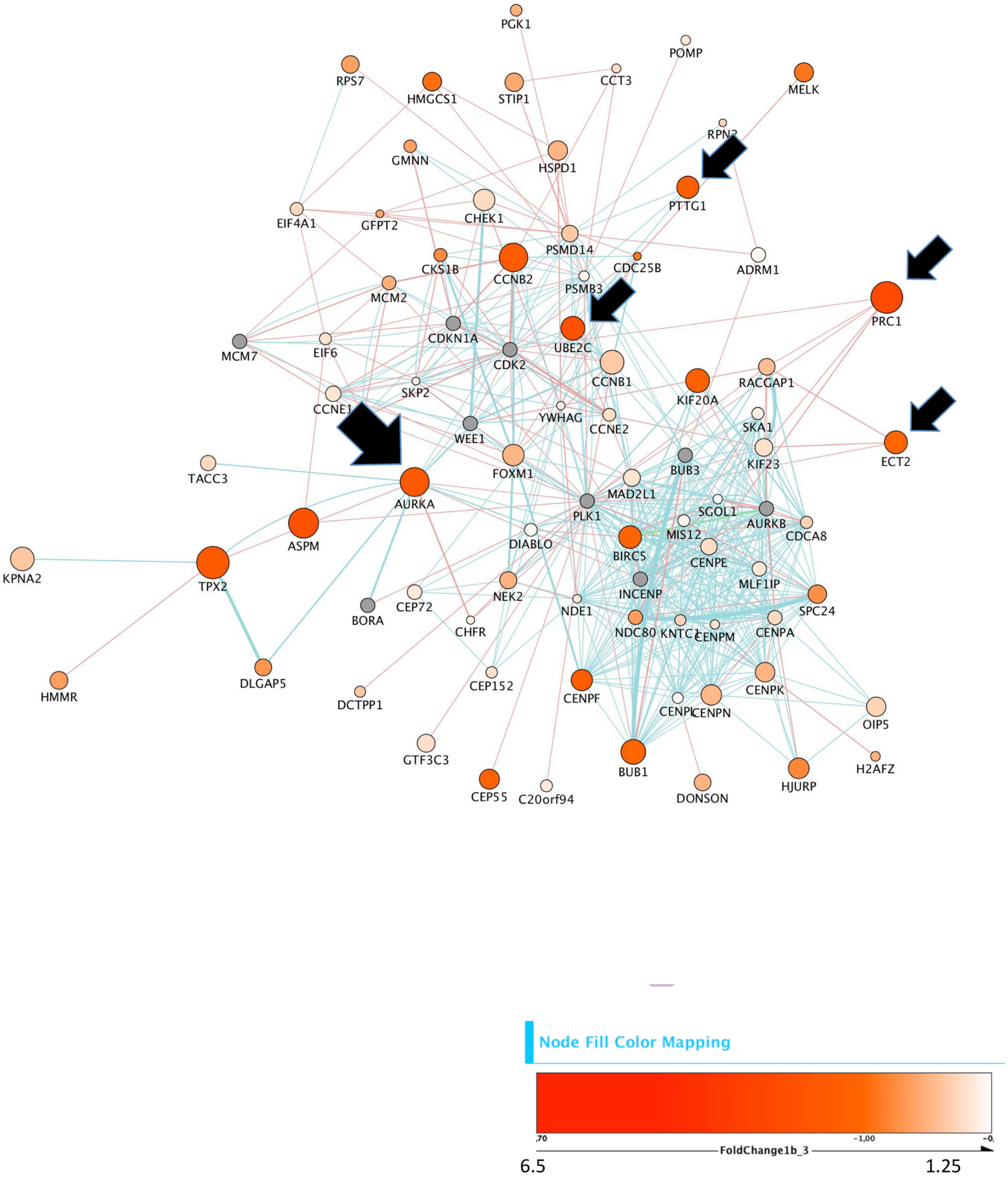

Supplementary Figure S8: Network of upregulated genes in WHO°III meningiomas related to AURKA. The network was generated by Genemania (http://www.genemania.org). Green Lines = genetic interactions, blue lines = pathways, red lines = physical interactions. Upregulated genes in WHO°III with p < 0.001 and a FC > 1.25 were used. The node color represents the fold change between WHO°III and 1NR. The node size in the graph anticorrelates with the p-value. Arrows = genes, which were chosen for qPCR validation.

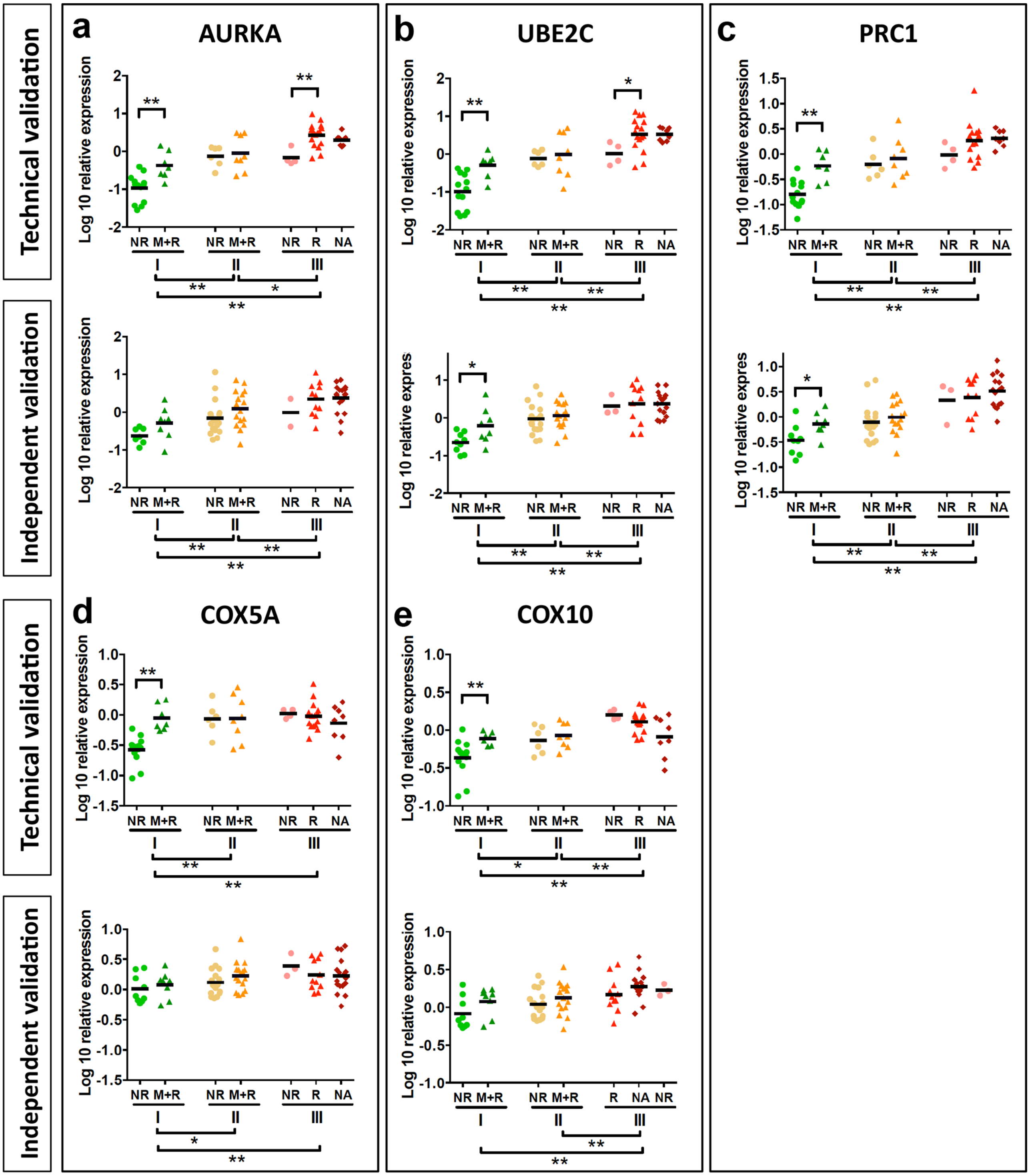

Supplementary Figure S9: Validation of candidate genes by qPCR. a. Analysis of *AURKA*, **b.** analysis of *UBE2C,* **c.** analysis of *PRC1,* **d.** analysis of *COX5A* and **e.** analysis of *COX10.* mRNA expression of candidate genes was analyzed in samples of the discovery set (a-e upper graph). A further validation was performed in an independent meningioma set (a–e lower graph). Mean mRNA expression of *AURKA, UBE2C, PRC1, COX5A* and *COX10* show a significant increase. Error bars represent mean of experiments done in triplicates. Statistical significance was determined by Mann-Whitney test, $*p < 0.05$, $**p < 0.01$.

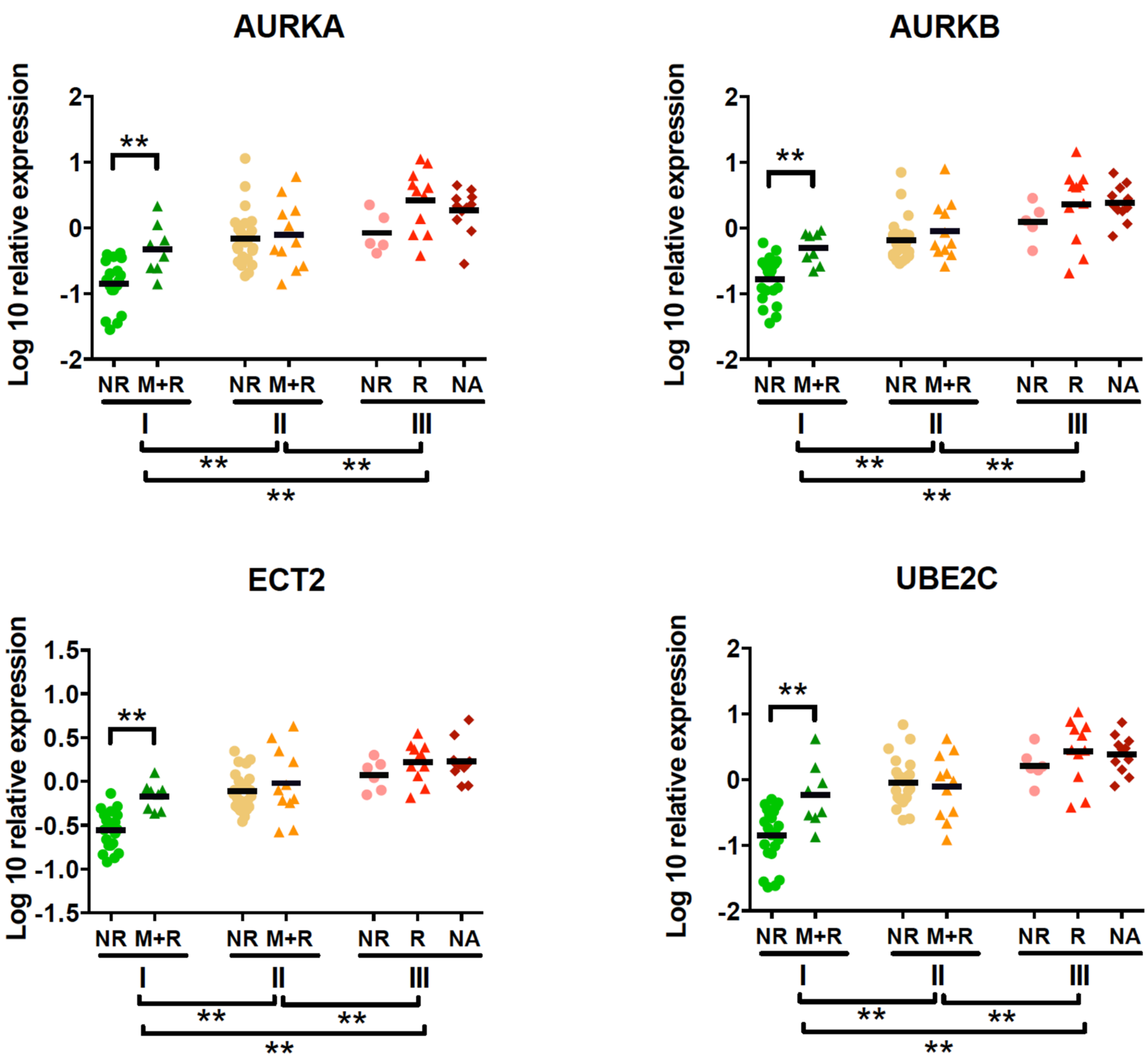

Supplementary Figure S10: qPCR validation in study sample including only newly diagnosed primary meningiomas. Microarray set and independent validation set were combined and only primary tumors were selected. Mean mRNA expression *of AURKA, AURKB, ECT2* and *UBE2C* shows a significant increase. Error bars represent mean of experiments done in triplicates. Statistical significance was determined by Mann-Whitney test, *p < 0.05, **p < 0.01.

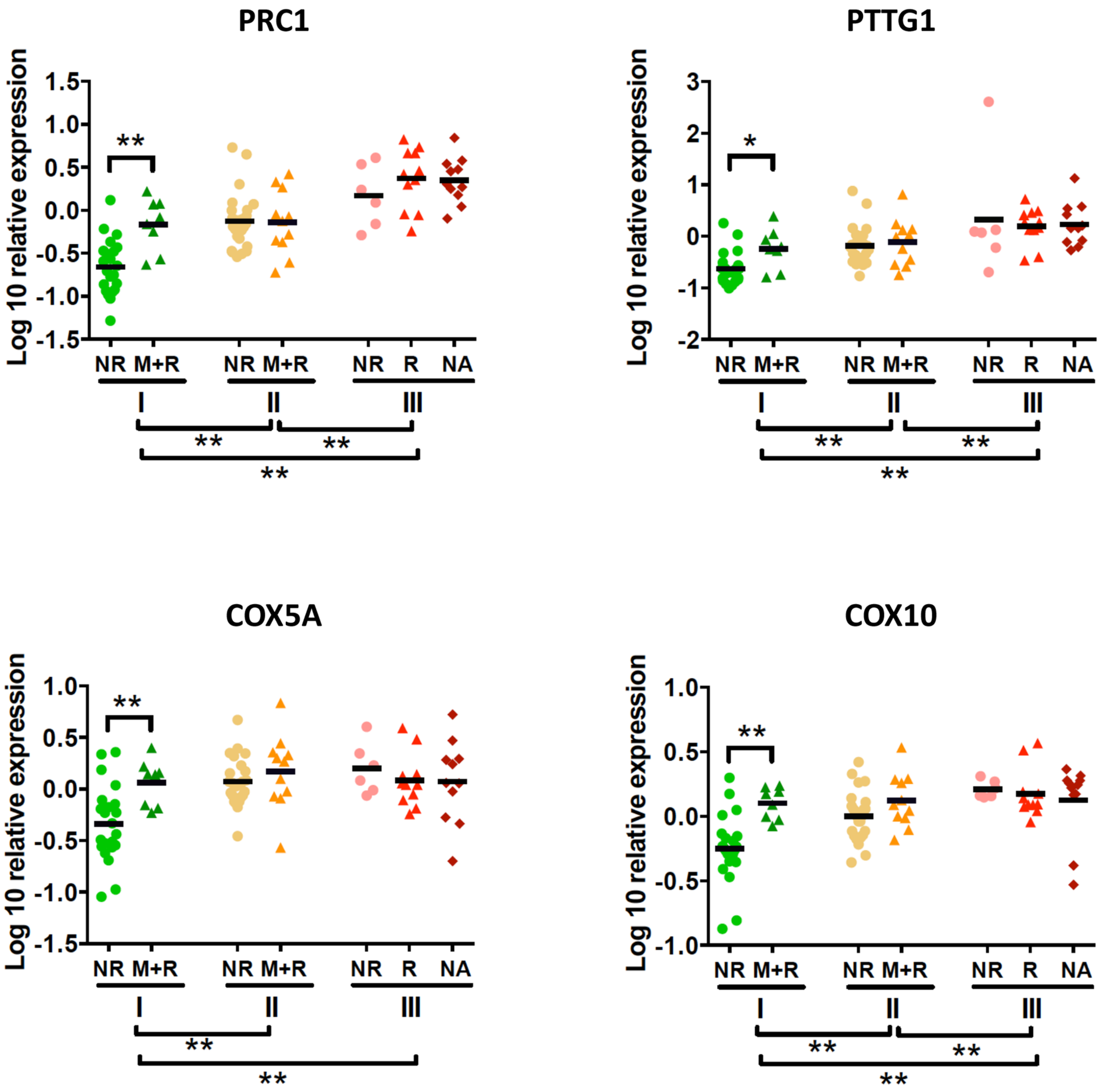

Supplementary Figure S11: qPCR validation in study sample including only newly diagnosed primary meningiomas. Microarray set and independent validation set were combined and only primary tumors were selected. Mean mRNA expression of *PRC1, PTTG1, COX5A* and *COX10* shows a significant increase. Error bars represent mean of experiments done in triplicates. Statistical significance was determined by Mann-Whitney test, $^*p < 0.05$, $^{**}p < 0.01$.

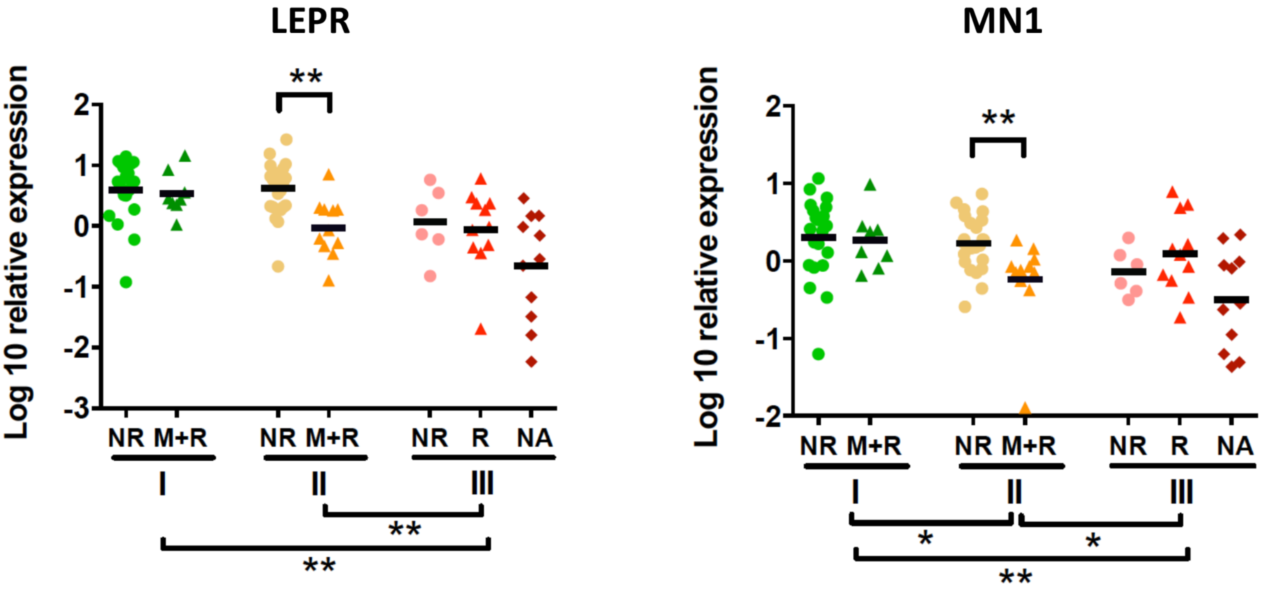

Supplementary Figure S12: qPCR validation in study sample including only newly diagnosed primary meningiomas. Microarray set and independent validation set were combined and only primary tumors were selected. Mean mRNA expression *LEPR* and *MN1* shows a significant decrease. Error bars represent mean of experiments done in triplicates. Statistical significance was determined by Mann-Whitney test, *p < 0.05, **p < 0.01.

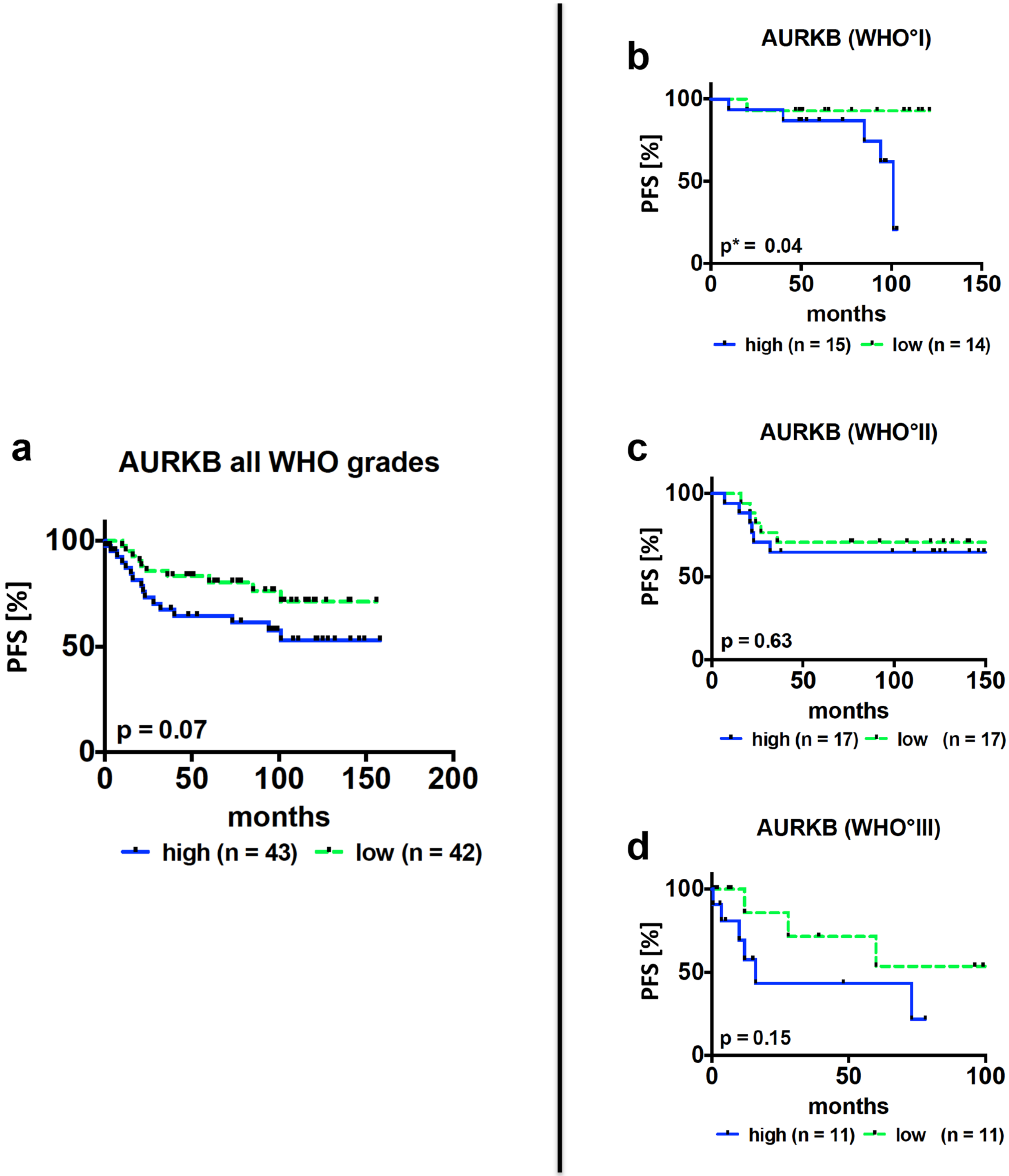

Supplementary Figure S13: Kaplan-Meier plots of progression free survival (PFS) related to *AURKB* expression. Only primary tumors and completely resected tumors (Simpson grade 1–3) were included. **a.** PFS of complete study sample (WHO°I–III) **b.** PFS of WHO°I **c.** PFS of WHO°II **d.** PFS of WHO°III. Patients were categorized into 2 groups according to the mean mRNA expression into high (blue curve) and low (green curve) expressions.

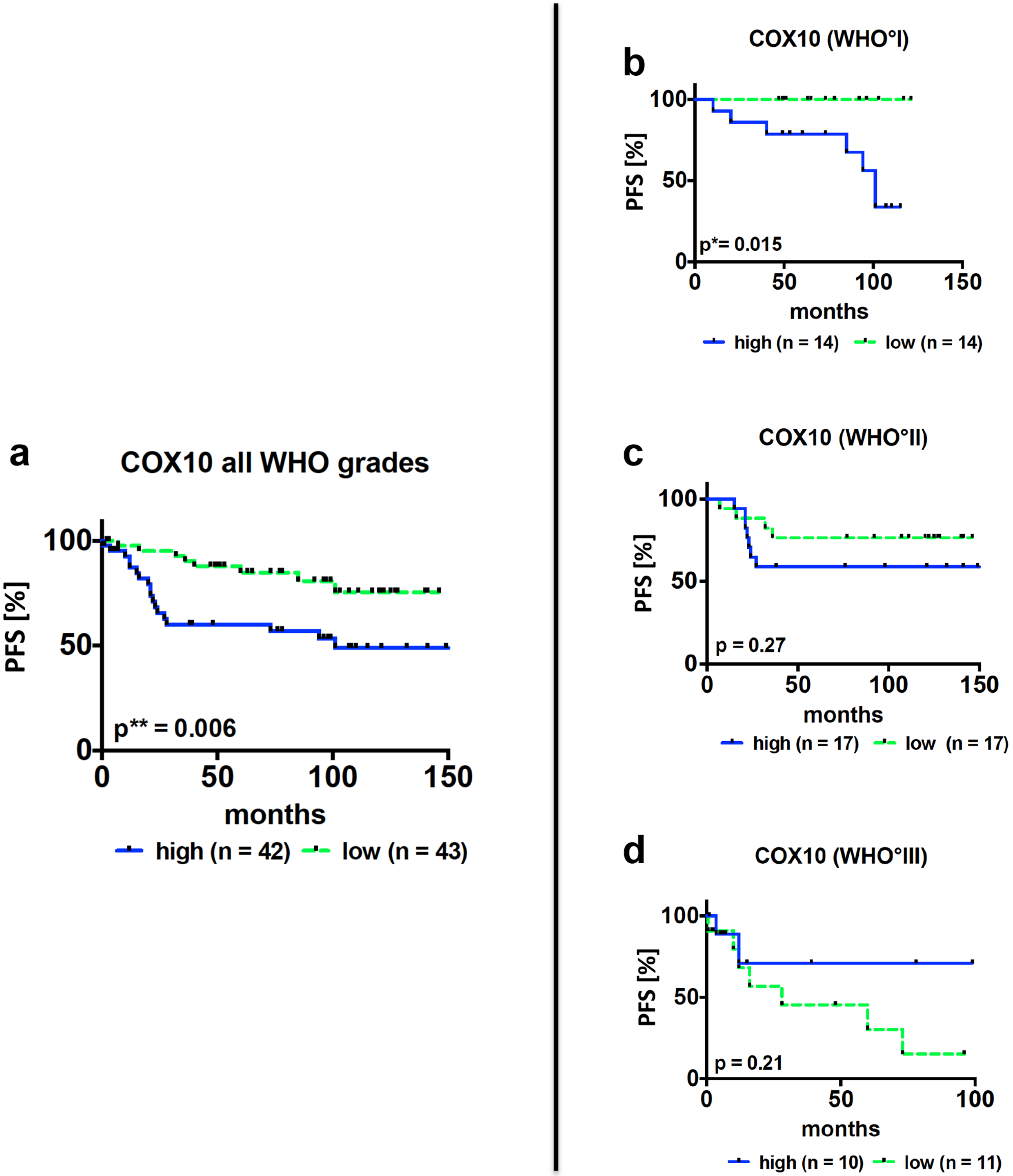

Supplementary Figure S14: Kaplan-Meier plots of progression free survival (PFS) related to *COX10* expression. Only primary tumors and completely resected tumors (Simpson grade 1–3) were included. **a.** PFS of complete study sample (WHO°I–III) **b.** PFS of WHO°I **c.** PFS of WHO°II **d.** PFS of WHO°III. Patients were categorized into 2 groups according to the mean mRNA expression into high (blue curve) and low (green curve) expressions.

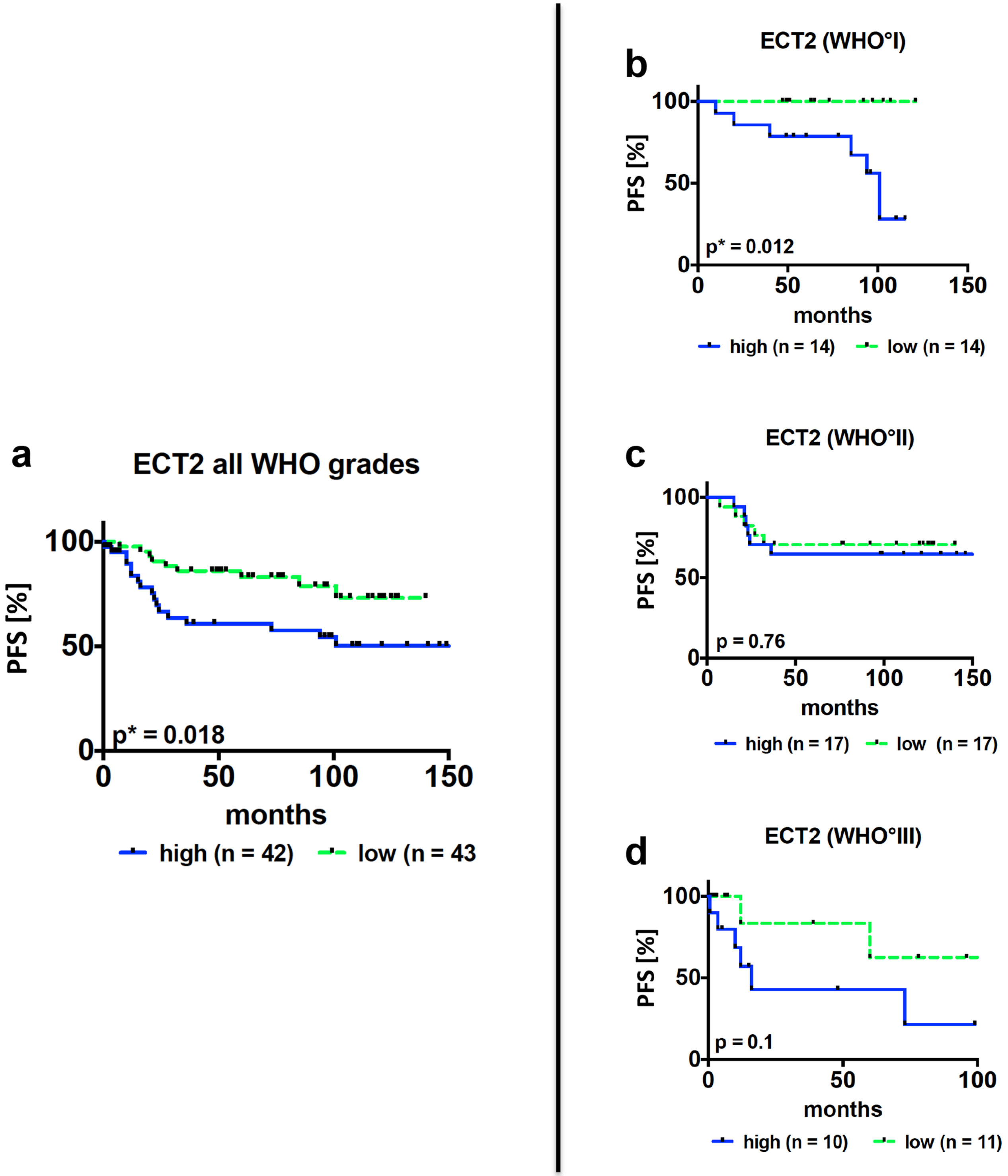

Supplementary Figure S15: Kaplan-Meier plots of progression free survival (PFS) related to *ECT2* expression. Only primary tumors and completely resected tumors (Simpson grade 1–3) were included. **a.** PFS of complete study sample (WHO°I–III) **b.** PFS of WHO°I **c.** PFS of WHO°II **d.** PFS of WHO°III. Patients were categorized into 2 groups according to the mean mRNA expression into high (blue curve) and low (green curve) expressions.

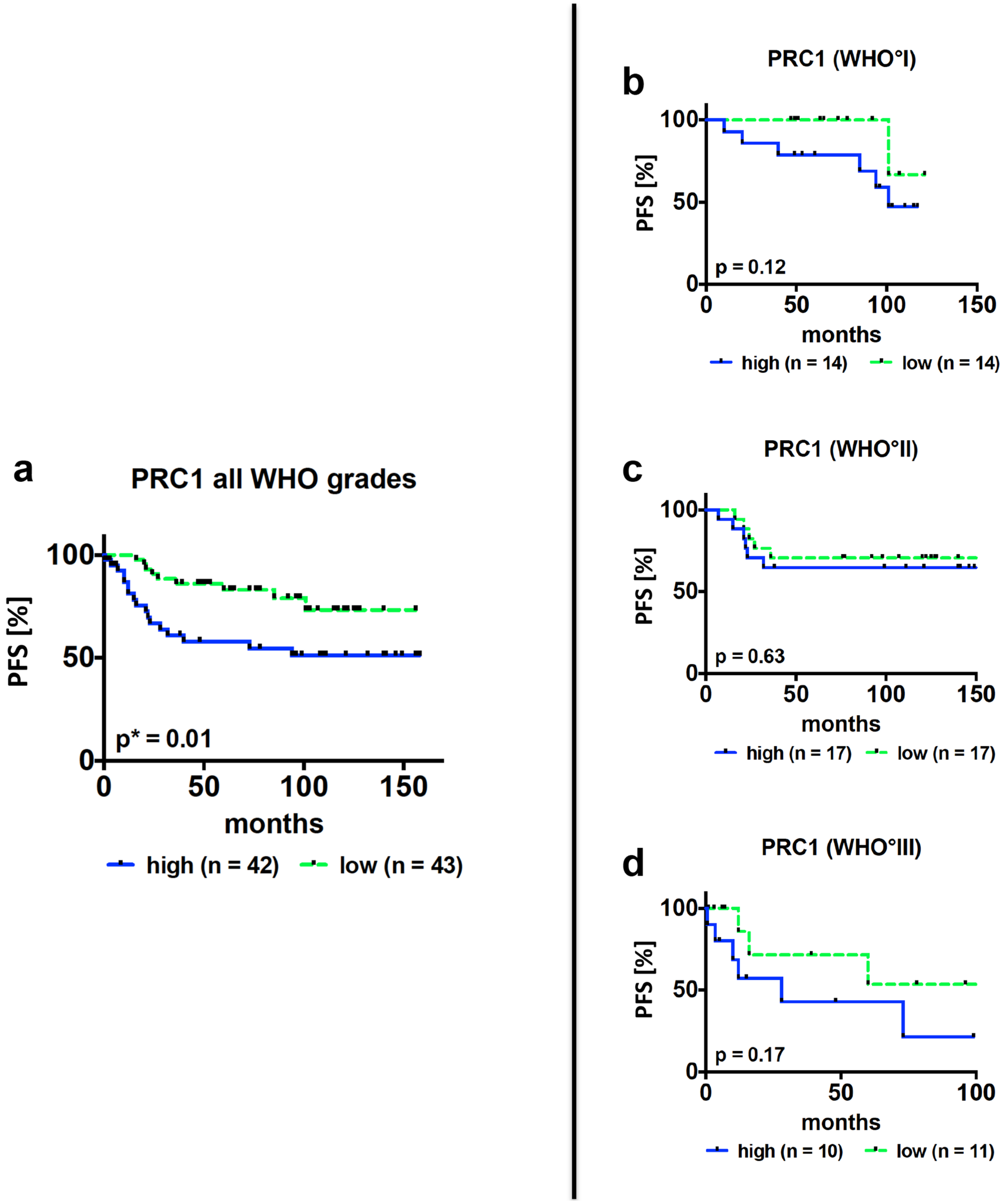

Supplementary Figure S16: Kaplan-Meier plots of progression free survival (PFS) related to *PRC1* expression. Only primary tumors and completely resected tumors (Simpson grade 1–3) were included. **a.** PFS of complete study sample (WHO°I–III) **b.** PFS of WHO°I **c.** PFS of WHO°II **d.** PFS of WHO°III. Patients were categorized into 2 groups according to the mean mRNA expression into high (blue curve) and low (green curve) expressions.

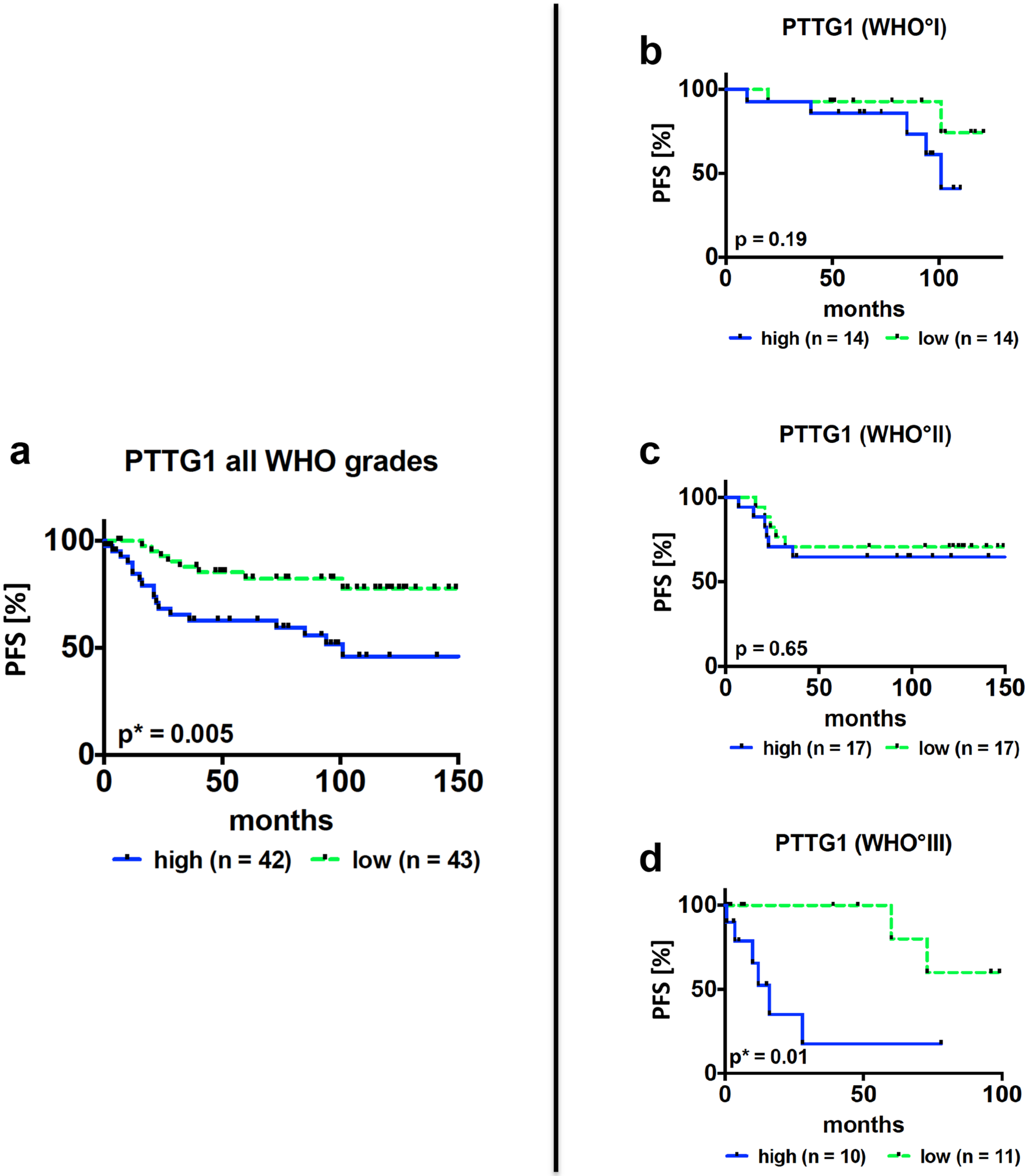

Supplementary Figure S17: Kaplan-Meier plots of progression free survival (PFS) related to *PTTG1* expression. Only primary tumors and completely resected tumors (Simpson grade 1–3) were included. **a.** PFS of complete study sample (WHO°I–III) **b.** PFS of WHO°I **c.** PFS of WHO°II **d.** PFS of WHO°III. Patients were categorized into 2 groups according to the mean mRNA expression into high (blue curve) and low (green curve) expressions.

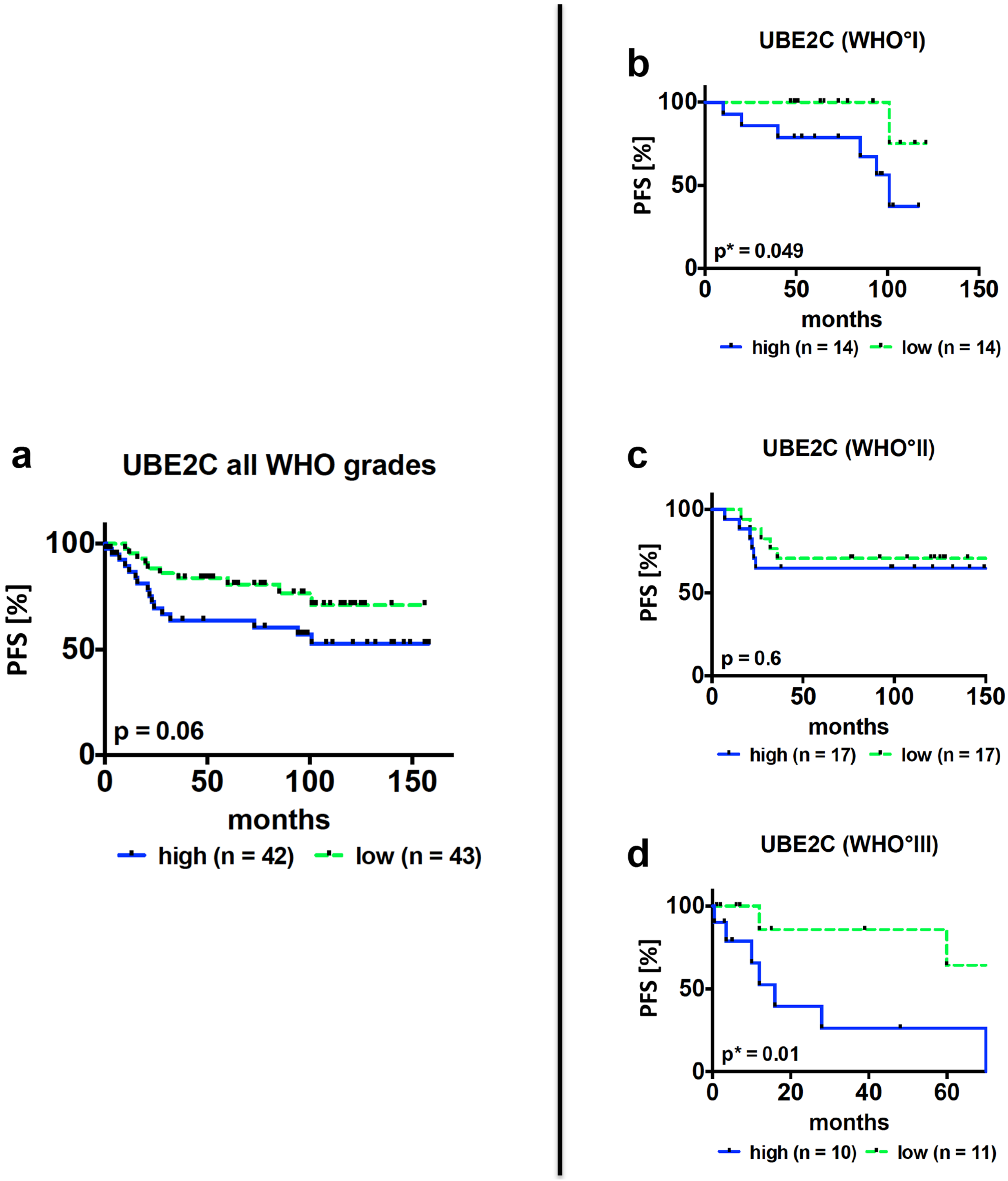

Supplementary Figure S18: Kaplan-Meier plots of progression free survival (PFS) related to *UBE2C* expression. Only primary tumors and completely resected tumors (Simpson grade 1–3) were included. **a.** PFS of complete study sample (WHO°I–III) **b.** PFS of WHO°I **c.** PFS of WHO°II **d.** PFS of WHO°III. Patients were categorized into 2 groups according to the mean mRNA expression into high (blue curve) and low (green curve) expressions.

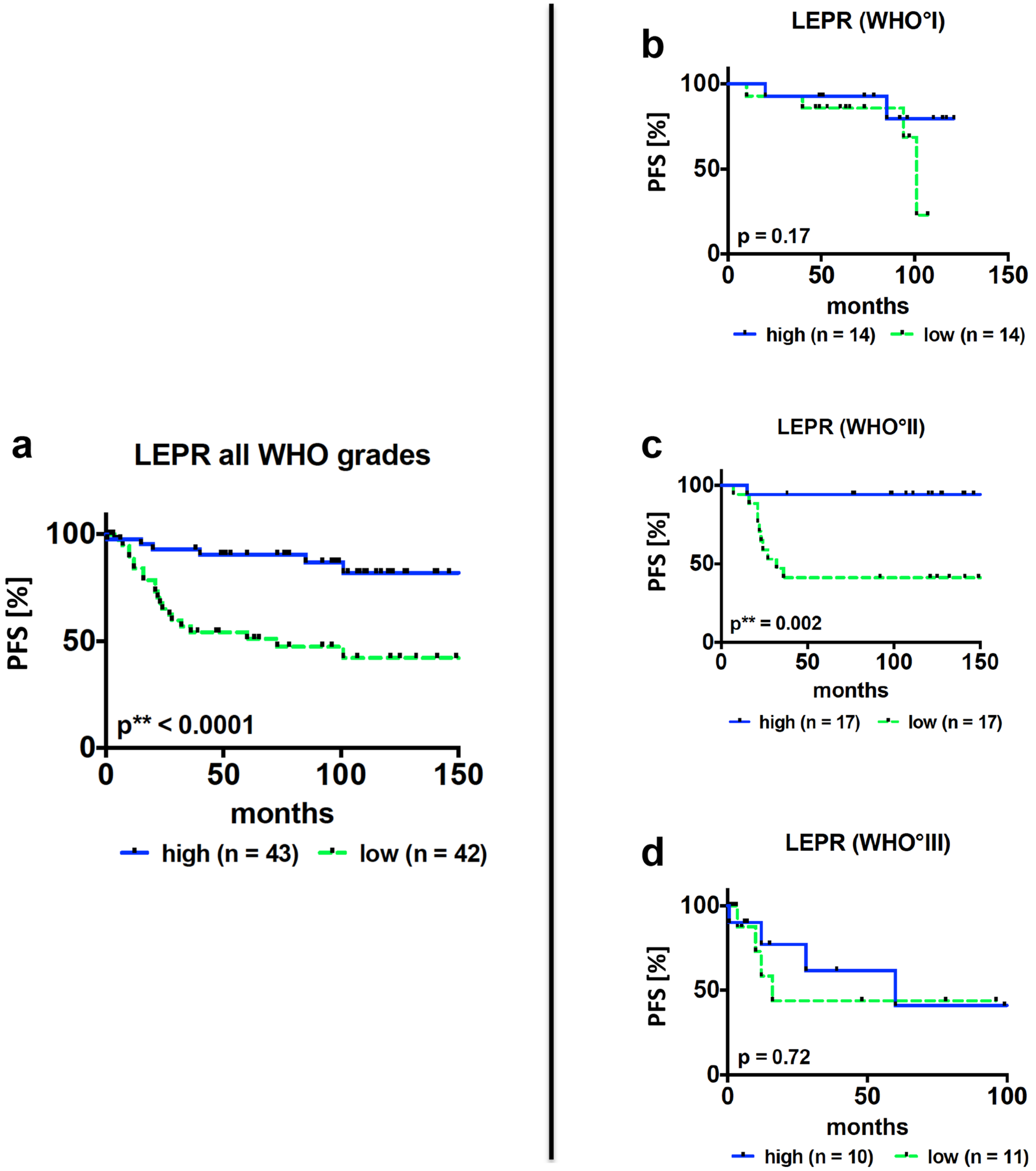

Supplementary Figure S19: Kaplan-Meier plots of progression free survival (PFS) related to *LEPR* expression. Only primary tumors and completely resected tumors (Simpson grade 1–3) were included. **a.** PFS of complete study sample (WHO°I–III) **b.** PFS of WHO°I **c.** PFS of WHO°II **d.** PFS of WHO°III. Patients were categorized into 2 groups according to the mean mRNA expression into high (blue curve) and low (green curve) expressions.

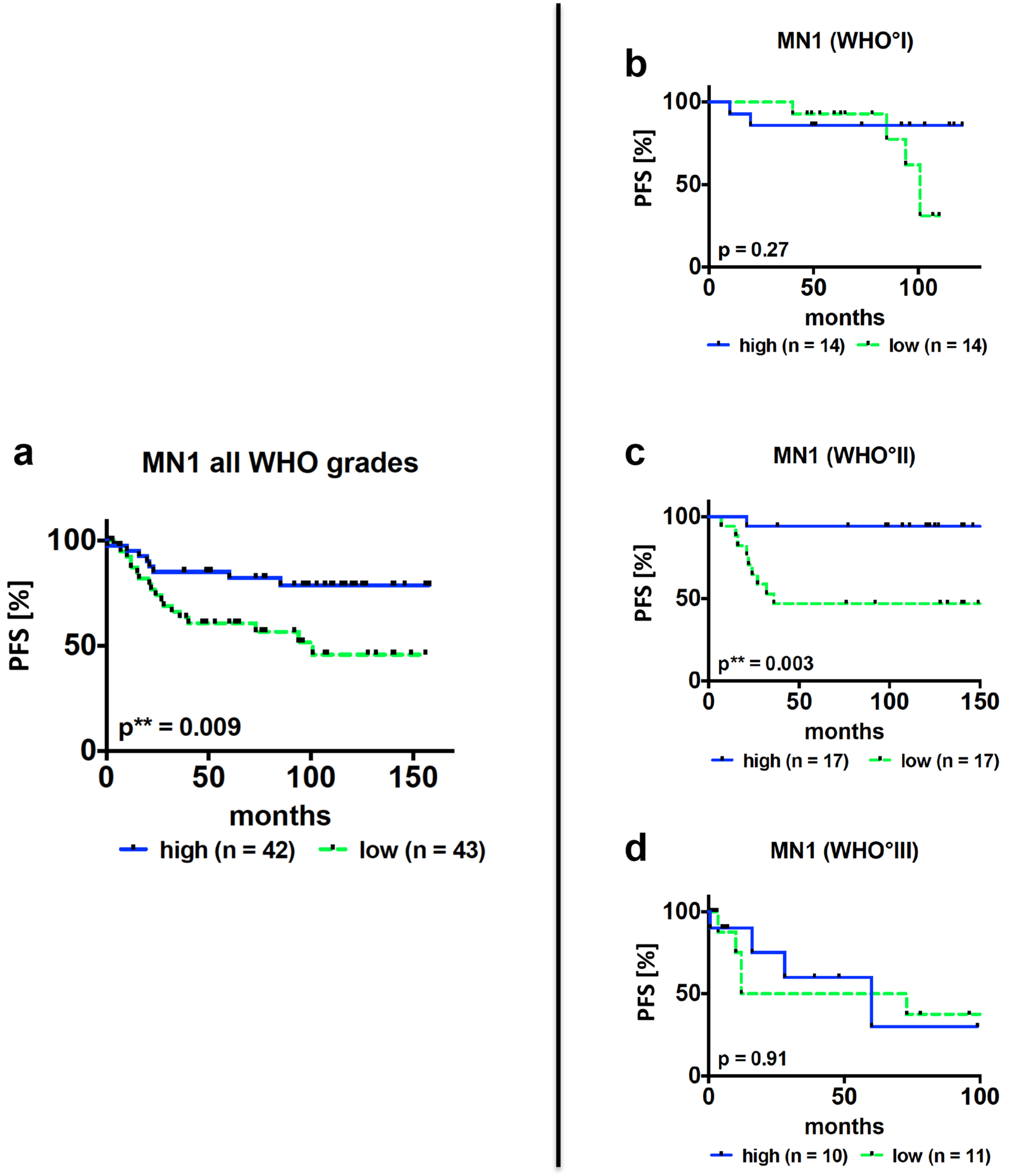

Supplementary Figure S20: Kaplan-Meier plots of progression free survival (PFS) related to *MN1* expression. Only primary tumors and completely resected tumors (Simpson grade 1–3) were included. **a.** PFS of complete study sample (WHO°I–III) **b.** PFS of WHO°I **c.** PFS of WHO°II **d.** PFS of WHO°III. Patients were categorized into 2 groups according to the mean mRNA expression into high (blue curve) and low (green curve) expressions.

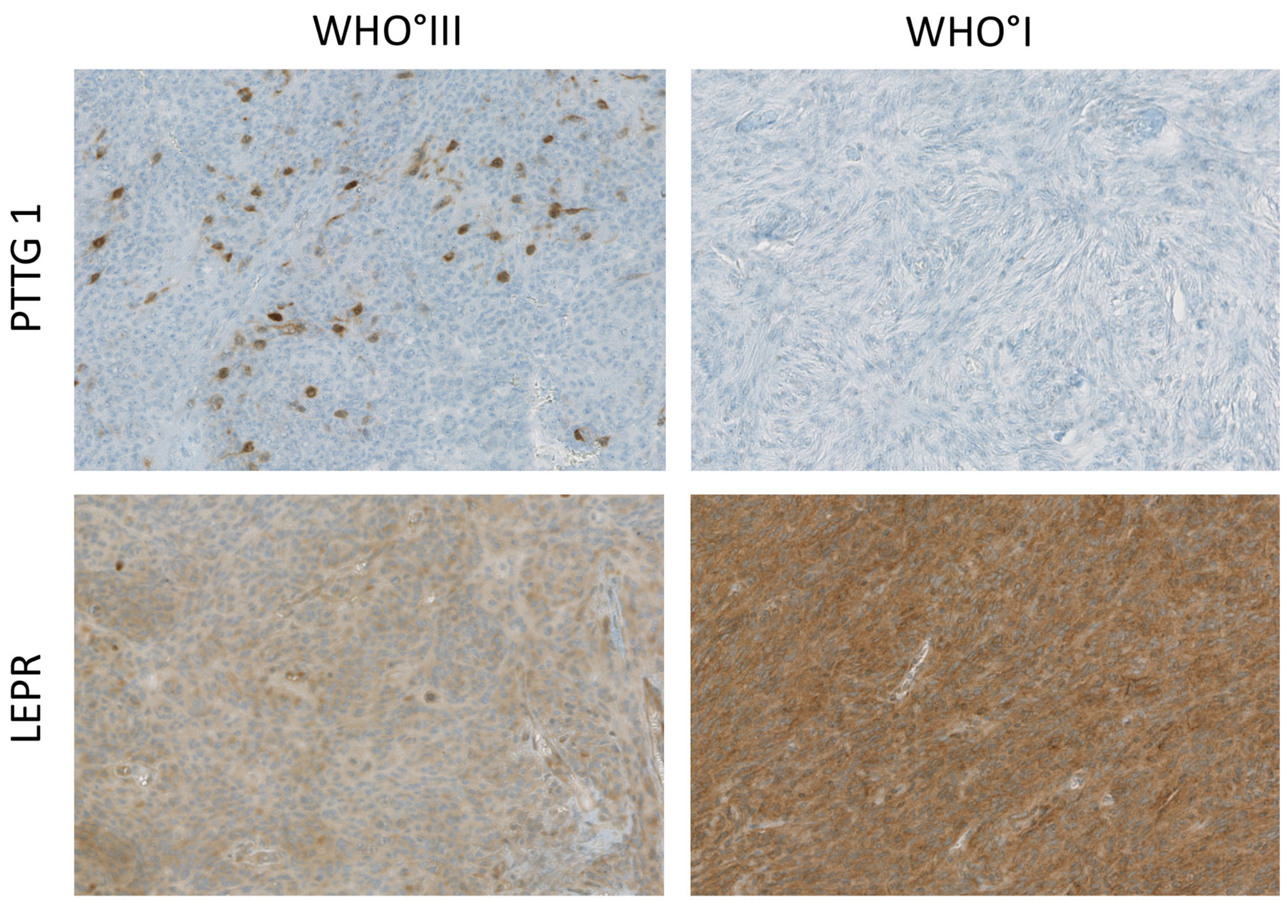

Supplementary Figure S21: Immunohistochemical staining of PTTG1 and LEPR. Pictures show representative samples of increased numbers of PTTG1-positive cells and reduced expression of LEPR in WHO grade III meningiomas.

Supplementary Table S1: Clinical and histological characteristics of meningiomas From treatment-naïve, primary, meningioma set (n = 87)

Characteristics	Primary treatment naive set	
	N	%
**Sex**		
Male	33	38
Female	54	62
**Age at 1st diagnosis [years]**		
Median	63	
Range	24 - 86	
**WHO Grade**		
WHO°I	29	33.3
WHO°II	35	40.2
WHO°III	23	26.4
**Subtype**		
Fibroblastic	8	9.2
Meningothelial	7	8
Transitional	12	13.8
Atypical	27	31
Angiomatous	0	0
Clear cell	1	1.1
Secretory	1	1.1
Anaplastic	11	12.6
Rhadoid	1	1.1
Papillary	2	2.3
Unknown	17	19.5
**Location**		
Frontal	4	4.6
Convexity	30	34.5
Falx	11	12.6
Parasagital	16	18.4
Skull base	17	19.6
Tentorial	3	3.4
Other	6	6.9
**Primary or recurrent tumor**		
Primary	87	100
Recurrent tumor	0	0

(*Continued*)

Characteristics	Primary treatment naive set	
	N	%
**Resection grade**		
Simpson° 1	53	61
Simpson° 2	18	20.6
Simpson° 1 or 2	2	2.3
Simpson° 3	14	16.1
Simpson° 4	0	0
Simpson° 5	0	0
**Postoperative treatment**		
Radiotherapy	22	25
Chemotherapy	0	0
**Clinical progression**		
Recurrence with same WHO°	22	25.3
Recurrence with higher WHO°	5	5.8
No recurrence	59	67.8
Unknown	1	1.1
**3-year progression-free time**		
WHO°I	27	93.1*
WHO°II	25	71.4*
WHO°III	9	39.1*
**3-year survival**		
WHO°I	28	96.6*
WHO°II	35	100*
WHO°III	10	43.5*
**Follow-up**	Median [months]	
WHO°I	92	
WHO°II	111	
WHO°III	27	

* = percentage of same WHO° tumors

Supplementary Table S2: Primer sequences from candidate genes

Gene name		Primer sequence (5' to 3')
AURKA #61	For	cgccctgtaggatactgctt
AURKA #61	Rev	caaatatccccgcactctg
AURKB #43	For	gatggcccagaaggagaact
AURKB #43	Rev	aggctctttccggaggact
PTTG1 #22	For	gcctctcatgatccttgacg
PTTG1 #22	Rev	gcttgaaggagactgcaaca
PRC1 #50	For	tttacaaaccgaggaggaaatc
PRC1 #50	Rev	tcgtgccttcaactcttcttc
BCL2L1 #66	For	agccttggatccaggagaa
BCL2L1 #66	Rev	agcggttgaagcgttcct
ECT2 #78	For	gtggttctggggaagcatt
ECT2 #78	Rev	aagcattgacactgatttcttgag
CAV2 #8	For	ccctcagctgtctgcacat
CAV2 #8	Rev	tcacactcttccatattgtctgc
COX5A #60	For	caaagtgtaaaccgcatggat
COX5A #60	Rev	tccaggtaactgttcacactcaa
COX10 #72	For	ggaaagagatgaagctgcaag
COX10 #72	Rev	tgcagtggtacttacaaccagag
CTSL2 #86	For	ggactctgaggaatcctatccat
CTSL2 #86	Rev	gcaacagaattctcaggtctgtact
MN1 #7	For	gacgacgacaagacgttgg
MN1 #7	Rev	gtttgcagggaggtcgtg
SERPINF1 #57	For	gtgtggagctgcagcgtat
SERPINF1 #57	Rev	tccaatgcagaggagtagca
LEPR #16	For	gtccttcctgattctgtggtg
LEPR #16	Rev	ttcccaagatattttcaataatcca
UBE2C #8	For	catgatgtctggcgataaagg
UBE2C #8	Rev	cgagagcttatacctcaggtcttc

For = forward, rev = reverse, # = number of TaqMan probe.

Supplementary Table S3: Supervised analysis of WHO grades and clinico-pathological subgroups

	Comparison	Number of genes (p<0.001)	FDR
**WHO°**	I vs II	124	0.145
	II vs III	99	0.172
	I vs III	1371	0.030
**Clinico-pathological subgroup**	1NR vs 1M	191	0.356
	1NR vs III	1764	0.015
	1M vs III	101	1.654
	2NR vs 2M+R	22	0.318
	1NR vs 2NR	443	0.075
	1NR vs 2M+R	587	0.291
	2NR vs 3	779	0.033
	1NR vs 2M+R	587	0.291
	1NR vs 1R	1291	0.035
	1NR vs 1M+R	464	0.099

False discovery rate between WHO grades and clinico-pathological subgroups using a supervised approach. Number of differentially expressed genes with a p-value < 0.001 are shown. FDR = False discovery rate, vs = versus, NR = non-recurrent, R = recurrent, M = malignantly progressing.

Supplementary Table S4: Real-time qPCR validation of 14 candidate genes identified by microarray analysis. Table shows results of technical validation for each gene between different clinicopathological subgroups. qPCR was performed as a technical validation in microarray training set. Colored fields show significant results. Dark red: significant higher expression with **p < 0.01; light red: significant higher expression with **p < 0.05; dark green: significant lower expression with **p < 0.01; light green: significant lower expression with **p < 0.05. NR = non-recurrent, R = recurrent, M = malignantly progressing, NA = no available clinical data for classification.

See Supplementary File 1.

Supplementary Table S5: List of differentially expressed genes shared between all aggressive meningioma subgroups independent of WHO grade as determined by the intersection study (n=332). Fold-changes and p-values for the respective groupings are given. A positive fold-change for e.g. the grouping 1NR_vs_1M+R equals a higher expression in group 1NR.

See Supplementary File 2.

Supplementary Table S6: Selected genes for qPCR validation

Gene for qPCR validation	Intersection studies	Pathway	Described in gene expression profiling of meningiomas	Described in tumor progression of other tumor entities
AURKA	y	Cell cycle	Y, but failed validation [19]	Y [6, 15, 7]
AURKB	y	Cell cycle	n	Y [6, 20]
BCL2L1	y		n	n
CAV2	y		n	Y [4]
COXA	y	Oxidat. phosphorylation	n	Y [2]
COX10	y	Oxidat. phosphorylation	n	n
CTSL2	y		n	Y [18]
ECT2	y		n	y [16, 17, 21, 22]
LEPR	y		Y [12]	n
MN1	y		(y) [10]	n
PRC1	y	Cell cycle	Y [1]	Y [13]
PTTG1	y	Cell cycle	n	Y [3, 21]
SERPINF1	y		Y [14]	n
UBE2C	y	Cell cycle	Y [5, 9]	Y [8, 11]

Candidate genes for qPCR validation were selected based on intersection studies, pathway enrichment analyses and literature research. Y = yes, n = no, oxidat. = oxidative.

REFERENCES

1. Bie L, Zhao G, Ju Y, Zhang B. Integrative genomic analysis identifies CCNB1 and CDC2 as candidate genes associated with meningioma recurrence. Cancer Genet 2011;204:536–540.
2. Chen W-L, Kuo K-T, Chou T-Y, Chen C-L, Wang C-H, Wei Y-H, Wang L-S. The role of cytochrome c oxidase subunit Va in non-small cell lung carcinoma cells: association with migration, invasion and prediction of distant metastasis. BMC Cancer 2012;12:273.
3. Demeure MJ, Coan KE, Grant CS, Komorowski RA, Stephan E, Sinari S, Mount D, Bussey KJ. PTTG1 overexpression in adrenocortical cancer is associated with poor survival and represents a potential therapeutic target. Surgery 2013;154:1405–1416.
4. Elsheikh SE, Green AR, Rakha EA, Samaka RM, Ammar AA, Powe D, Reis-Filho JS, Ellis IO. Caveolin 1 and Caveolin 2 are associated with breast cancer basal-like and triple-negative immunophenotype. Br J Cancer 2008;99:327–334.
5. Fèvre-Montange M, Champier J, Durand A, Wierinckx A, Honnorat J, Guyotat J, Jouvet A. Microarray gene expression profiling in meningiomas: differential expression according to grade or histopathological subtype. Int J Oncol 2009;35:1395–1407.
6. Goldenson B, Crispino JD. The aurora kinases in cell cycle and leukemia. Oncogene 2015;34:537–545.
7. Goos JACM, Coupe VMH, Diosdado B, Delis-Van Diemen PM, Karga C, Beliën JAM, Carvalho B, van den Tol MP, Verheul HMW, Geldof AA, Meijer GA, Hoekstra OS, Fijneman RJA, et al. Aurora kinase A (AURKA) expression in colorectal cancer liver metastasis is associated with poor prognosis. Br J Cancer 2013;109:2445–2452.
8. Hao Z, Zhang H, Cowell J. Ubiquitin-conjugating enzyme UBE2C: molecular biology, role in tumorigenesis, and potential as a biomarker. Tumour Biol 2012;33:723–730.
9. Jiang L, Wang T, Bao Y, Qian J, Wu X-J, Hu G-H, Lu Y-C. A study of UbcH10 expression and its association with recurrence of meningiomas. J Surg Oncol 2012;106:327–331.

10. Lekanne Deprez RH, Riegman PH, Groen NA, Warringa UL, van Biezen NA, Molijn AC, Bootsma D, de Jong PJ, Menon AG, Kley NA. Cloning and characterization of MN1, a gene from chromosome 22q11, which is disrupted by a balanced translocation in a meningioma. Oncogene 1995;10:1521–1528.
11. Loussouarn D, Campion L, Leclair F, Campone M, Charbonnel C, Ricolleau G, Gouraud W, Bataille R, Jézéquel P. Validation of UBE2C protein as a prognostic marker in node-positive breast cancer. Br J Cancer 2009;101:166–173.
12. Menghi F, Orzan FN, Eoli M, Farinotti M, Maderna E, Pisati F, Bianchessi D, Valletta L, Lodrini S, Galli G, Anghileri E, Pellegatta S, Pollo B, et al. DNA microarray analysis identifies CKS2 and LEPR as potential markers of meningioma recurrence. Oncologist 2011;16:1440–1450.
13. Pérez de Castro I, Malumbres M. Mitotic Stress and Chromosomal Instability in Cancer: The Case for TPX2. Genes Cancer 2012;3:721–730.
14. Pérez-Magán E, Rodríguez de Lope A, Ribalta T, Ruano Y, Campos-Martín Y, Pérez-Bautista G, García JF, García-Claver A, Fiaño C, Hernández-Moneo J-L, Mollejo M, Meléndez B. Differential expression profiling analyses identifies downregulation of 1p, 6q, and 14q genes and overexpression of 6p histone cluster 1 genes as markers of recurrence in meningiomas. Neuro-oncology 2010;12:1278–1290.
15. Regan JL, Sourisseau T, Soady K, Kendrick H, McCarthy A, Tang C, Brennan K, Linardopoulos S, White DE, Smalley MJ. Aurora A kinase regulates mammary epithelial cell fate by determining mitotic spindle orientation in a Notch-dependent manner. Cell Rep 2013;4:110–123.
16. Saito S, Liu X-F, Kamijo K, Raziuddin R, Tatsumoto T, Okamoto I, Chen X, Lee C-C, Lorenzi MV, Ohara N, Miki T. Deregulation and mislocalization of the cytokinesis regulator ECT2 activate the Rho signaling pathways leading to malignant transformation. J Biol Chem 2004;279:7169–7179.
17. Sano M, Genkai N, Yajima N, Tsuchiya N, Homma J, Tanaka R, Miki T, Yamanaka R. Expression level of ECT2 proto-oncogene correlates with prognosis in glioma patients. Oncol Rep 2006;16:1093–1098.
18. Skrzypczak M, Springwald A, Lattrich C, Häring J, Schüler S, Ortmann O, Treeck O. Expression of cysteine protease cathepsin L is increased in endometrial cancer and correlates with expression of growth regulatory genes. Cancer Invest 2012;30:398–403.
19. Stuart JE, Lusis EA, Scheck AC, Coons SW, Lal A, Perry A, Gutmann DH. Identification of gene markers associated with aggressive meningioma by filtering across multiple sets of gene expression arrays. J Neuropathol Exp Neurol 2011;70:1–12.
20. Vasiljevic A, Champier J, Figarella-Branger D, Wierinckx A, Jouvet A, Fèvre-Montange M. Molecular characterization of central neurocytomas: potential markers for tumor typing and progression. Neuropathology 2013;33:149–161.
21. Wondergem B, Zhang Z, Huang D, Ong CK, Koeman J, Hof DV, Petillo D, Ooi A, Anema J, Lane B, Kahnoski RJ, Furge KA, Teh BT. Expression of the PTTG1 oncogene is associated with aggressive clear cell renal cell carcinoma. Cancer Res 2012;72:4361–4371.
