# Supplementary material for: Transcriptomic analysis of aggressive meningiomas identifies PTTG1 and LEPR as prognostic biomarkers independent of WHO grade
Source: Oncotarget. 2016 Feb 15;7(12):14551–68. doi: 10.18632/oncotarget.7396 (PMC4924735; doi:10.18632/oncotarget.7396)
Supplement: Supplementary file 2 [file oncotarget-07-14551-s002.docx]

| **Comparison** | **III vs I** | **III vs II** | **II vs I** | **III vs 1NR** | **2M+R vs 1NR** | **1M+R vs 1NR** | **2M+R vs 2NR** | **3R vs 3NR** |
| --- | --- | --- | --- | --- | --- | --- | --- | --- |
| **PTTG1** |  |  |  |  |  |  |  |  |
| **AURKA** |  |  |  |  |  |  |  |  |
| **AURKB** |  |  |  |  |  |  |  |  |
| **BCL2L1** |  |  |  |  |  |  |  |  |
| **CAV2** |  |  |  |  |  |  |  |  |
| **COX5A**  **Candidate**  **genes** |  |  |  |  |  |  |  |  |
| **COX10** |  |  |  |  |  |  |  |  |
| **CTSL2** |  |  |  |  |  |  |  |  |
| **ECT2** |  |  |  |  |  |  |  |  |
| **PRC1** |  |  |  |  |  |  |  |  |
| **UBE2C** |  |  |  |  |  |  |  |  |
| **LEPR** |  |  |  |  |  |  |  |  |
| **MN1** |  |  |  |  |  |  |  |  |
| **SERPINF1** |  |  |  |  |  |  |  |  |
